# Molecular Mechanism of pH-Induced Protrusion Configuration Switching in Piscine Betanodavirus Implies a Novel Antiviral Strategy

**DOI:** 10.1021/acsinfecdis.4c00407

**Published:** 2024-08-01

**Authors:** Petra Štěrbová, Chun-Hsiung Wang, Kathleen J. D. Carillo, Yuan-Chao Lou, Takayuki Kato, Keiichi Namba, Der-Lii M. Tzou, Wei-Hau Chang

**Affiliations:** † Chemical Biology and Molecular Biophysics Program, Taiwan International Graduate Program, Academia Sinica, Taipei 11529, Taiwan; ‡ College of Life Science, National Tsing Hua University, Hsinchu 30044, Taiwan; § Institute of Chemistry, Academia Sinica, Taipei 11529, Taiwan; ∥ Biomedical Translation Research Center, Academia Sinica, Taipei 11529, Taiwan; ⊥ Graduate School of Frontier Biosciences, Osaka University, 1-3 Yamadaoka, Suita, Osaka 565-0871, Japan; # Genomics Research Center, Academia Sinica, Taipei 11529, Taiwan; ∇ Institute of Physics, Academia Sinica, Taipei 11529, Taiwan

**Keywords:** conformational change, druggable pocket, protein oligomerization, pH sensing, disorder-to-order transition, solution structures, molecular movies

## Abstract

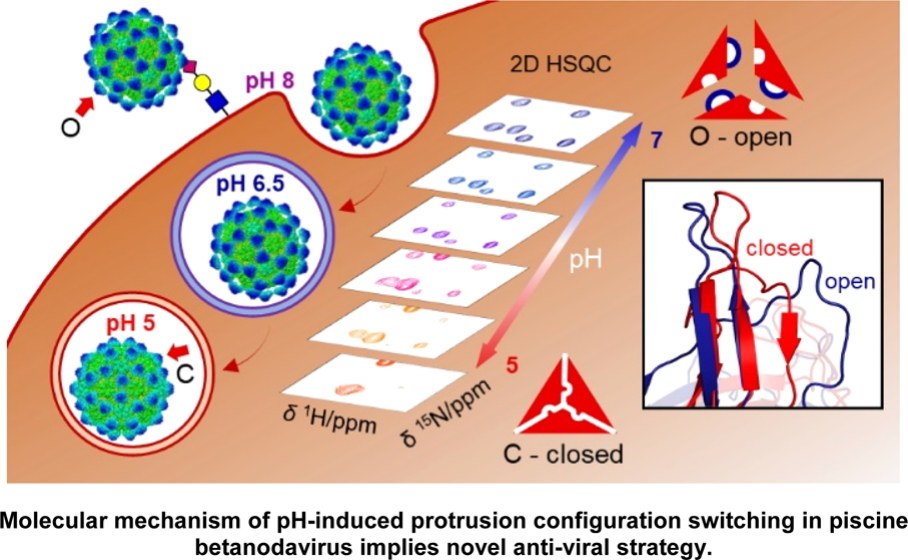

Many viruses contain surface spikes or protrusions that are essential for virus entry. These surface structures can thereby be targeted by antiviral drugs to treat viral infections. Nervous necrosis virus (NNV), a simple nonenveloped virus in the genus of betanodavirus, infects fish and damages aquaculture worldwide. NNV has 60 conspicuous surface protrusions, each comprising three protrusion domains (P-domain) of its capsid protein. NNV uses protrusions to bind to common receptors of sialic acids on the host cell surface to initiate its entry via the endocytic pathway. However, structural alterations of NNV in response to acidic conditions encountered during this pathway remain unknown, while detailed interactions of protrusions with receptors are unclear. Here, we used cryo-EM to discover that Grouper NNV protrusions undergo low-pH-induced compaction and resting. NMR and molecular dynamics (MD) simulations were employed to probe the atomic details. A solution structure of the P-domain at pH 7.0 revealed a long flexible loop (amino acids 311–330) and a pocket outlined by this loop. Molecular docking analysis showed that the N-terminal moiety of sialic acid inserted into this pocket to interact with conserved residues inside. MD simulations demonstrated that part of this loop converted to a β-strand under acidic conditions, allowing for P-domain trimerization and compaction. Additionally, a low-pH-favored conformation is attained for the linker connecting the P-domain to the NNV shell, conferring resting protrusions. Our findings uncover novel pH-dependent conformational switching mechanisms underlying NNV protrusion dynamics potentially utilized for facilitating NNV entry, providing new structural insights into complex NNV-host interactions with the identification of putative druggable hotspots on the protrusion.

Viral infections remain a major global health and economic threat.^1,2^ Viruses are virtually nanoscale organic materials that can infect and replicate themselves inside host cells by using their own or host cell’s replication apparatus.^3^ During an infection cycle, the viral genome encapsulated inside a protein capsid is delivered to a designated site in the host cell where it can be replicated, prior to which the viral genome must be released from the protein capsid. This process is usually enabled by profound structural alterations of the viral capsid induced by external stimuli, such as low pH, ligand binding, or interaction with the cell receptor.^4−7^ Investigating structures of a virus capsid and their induced changes is crucial for understanding the pathological process and may offer clues to design antiviral drugs or vaccines.

Nervous necrosis virus (NNV) has emerged as one of the most widespread fish viruses to threat fish farming worldwide.^2,8^ Outbreaks of the viral neuropathies and retinopathies caused by NNV result in almost 100% mortality of infected stock, with larval and juvenile fish being the most susceptible to NNV infections. Since the first characterization of the virus from larval striped jack (*Pseudocaranx dentex*) in the 1990s,^9^ NNV infections have been reported in more than 120 marine and freshwater fish.^10^ Due to the lack of an effective vaccine, NNV infections continue to severely damage the aquacultural economy.

NNV is a small nonenveloped RNA virus in the Genus *Betanodavirus* and Family *Nodaviridae*. In *Nodaviridae*, there are two genera: alphanodaviruses primarily infecting insects and betanodaviruses whose natural hosts are fish.^11,12^ An NNV particle encapsulates two positive-sense single-stranded RNA molecules: RNA1 (3.1 kilobases, kb) encodes an RNA-dependent RNA polymerase, whereas RNA2 (1.4 kb) encodes a single structural capsid protein (CP).^11^ A subgenomic RNA (RNA3), comprising part of the 3′ end of RNA1, has been identified as encoding a nonstructural protein B2 acting as an antagonist to host RNA interference.^13^

NNV has been demonstrated to enter host cells primarily via endocytic pathway.^14−16^ During this process, viruses are internalized into host cells via small vesicles, eventually arriving at late endosomes or endolysosomes for their destruction. The decrease of the pH inside an endosome caused by endosome acidification may serve as a trigger for the release of the virus genome into the host cytoplasma,^17−19^ as exemplified by Flock House Virus (FHV), a nonenveloped virus in Genus Alphanodavirus. Exposure of FHV to low endocytic pH induces structural changes of the FHV particle and release of its membrane-interacting γ peptide (4.4 kDa) for disrupting the endosomal membrane.^20,21^ Although details of low-pH-induced changes during endocytosis are well-described for FHV, those mechanisms are unlikely to be applicable to betanodaviruses since not only the CP sequences of alphanodaviruses and betanodaviruses share low similarity but also the capsid structures of the two differ markedly—unlike betanodavirus, alphanodaviruses do not have conspicuous surface protrusions.^12^ These protrusions are evidently powerful molecular structures used by betanodavirus to invade host cells.

A high-resolution structure of NNV capsid was obtained from crystals of Grouper NNV (GNNV) virus-like particles (VLPs) grown in a neutral condition (pH 7.2) that diffracted to near atomic resolution (3.6 Å, PDB 4WIZ).^22^ The conditions used for GNNV VLP crystal growth are similar to those when the virus attaches to the host cell. The structure of GNNV VLP unveils GNNV capsid is a *T* = 3 icosahedral particle with a diameter of ∼35 nm and delineates the CP as consisting of three domains: an N-terminal arm, a shell domain (S-domain), and a protrusion domain (P-domain). The S-domain comprises an eight-stranded β-sheet to form a canonical “jelly-roll” structure as the building unit of virus capsid; three P-domains from contiguous CPs in one asymmetric unit are located outside of the capsid, presenting a protrusion at the quasi-3-fold axis. As the P-domain is connected to the S-domain via a flexible linker and thereby not well-resolved, it has been separately expressed and crystallized in a weakly acidic condition (pH 6.5) with an overwhelming amount of calcium ions (0.2 M). The structure of the isolated P-domain was resolved to an atomic resolution (1.2 Å, PDB 4RFU). In the crystal, three P-domain molecules assemble into a compact trimer. Notably, the trimer in the crystal is stabilized by two calcium ions coordinated by three sets of _273_DxD_275_ motifs from neighboring subunits. As NNV protrusions play a central role in NNV infectivity^23,24^ and determining NNV host specificity,^25−27^ this crystal structure^22^ of the P-domain has become the structural basis for understanding NNV infections. Accordingly, the configuration of the protrusion is believed to be compact and static when NNV engages with a host receptor.^28^ However, comparison of a high-quality cryo-EM map of GNNV VLP generated previously^29^ against the respective crystal structure^22^ shows that the protrusions may gain dynamics as the virus particles are in solution.

As cryo-EM has advantages over X-ray crystallography in easily accessing to solution conformations^30^ and aqueous conditions mimicking physiological environments with changes in pH, ions,^31^ or temperature,^32^ we herein used cryo-EM to investigate the pH dependence of GNNV structures. The aqueous conditions mimicking those during the route of endocytosis were used to embed GNNV particles prior to their freezing. Specifically, we examined three pH points: weakly alkaline (pH 8.0) for host cell docking, weakly acidic (pH 6.5) in the early endosome, and acidic (pH 5.0) in the late endosome. Our cryo-EM results show the protrusions on GNNV adopt erect/loose configuration at pH 8.0 and 6.5 but become prone/compact at pH 5.0. To delve into the atomic details underlying such dynamics, we separately expressed the GNNV P-domain for solution analysis. Strikingly, we found the P-domain adopted a monomeric form at pH 7.0 but could convert to a trimer at pH 5.0. Further NMR structural determinations of the P-domain (pH 7.0) yielded the first solution structure of the P-domain, identifying a region of 20 amino acids (aa; 311–330) that adopts a long flexible loop, contrasting with it being interrupted by a short β-strand (aa 323–326) in the crystal structure.^22^ We used this solution structure as the basis for molecular dynamics (MD) simulations and discovered that this loop region can undergo acid-induced disorder-to-order transitions, inducing the P-domain to self-assemble into a compact trimer. In this study, we demonstrate that the NNV capsid acts as a pH-responsive nanoscale machine and unravels the molecular mechanism underlying the pH-dependent dynamics of GNNV protrusions. These findings provide new structural insights into the operation of NNV during its infection and imply druggable hotspots on it for guiding structure-based design of vaccines or inhibitors against NNVs.

## Results

### Cryo-EM Reveals That GNNV Protrusions Are Dynamic Entities

**Figure 1:**
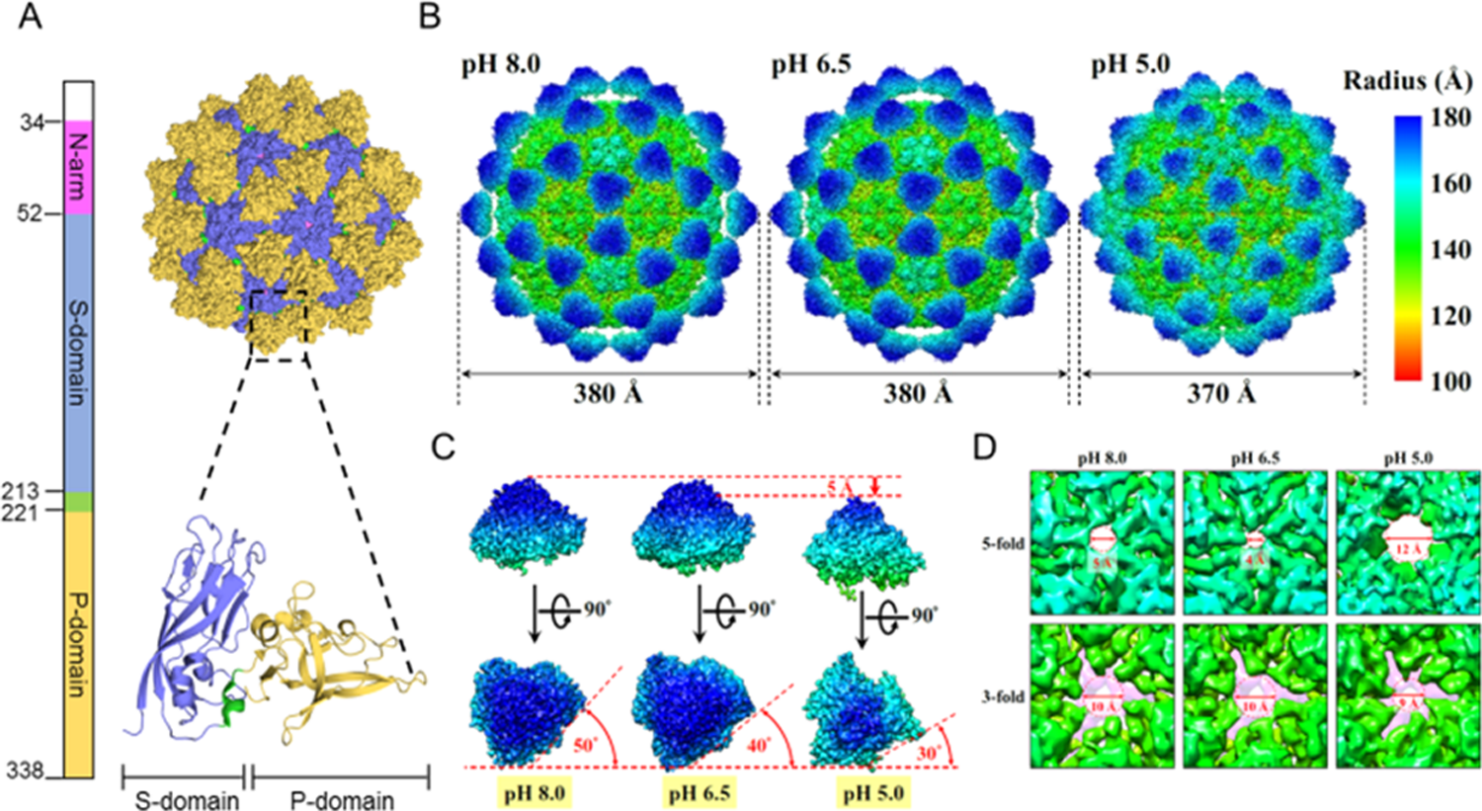
GNNV atomic model and cryo-EM structures in three different pH environments. (A) Atomic models of GNNV S-domain (PDB 4WIZ) and P-domain (PDB 4RFU). (B) Surface views of GNNV virus-like particles at pH 8.0 (*left panel*), 6.5 (*middle panel*), and 5.0 (*right panel*). The views are colored with a heatmap according to the local radius. (C) Conformational changes of the protrusion in response to the change in pH: 8.0 (*left panel*), 6.5 (*middle panel*), and 5.0 (*right panel*). The protrusions rotate clockwise and shift ∼5 Å toward the capsid shell as the pH decreases. (D) Enlarged views of pores (at 5- and 3-fold axis) in the capsid shell surface for different pH environments. The size of a pore at each pH condition is indicated. To highlight the pore at the 3-fold axis, the densities underneath are colored in light purple.

Close inspection of a high-quality cryo-EM map of a GNNV VLP^29^ against the crystal structure^22^ indicates that the protrusions adopt a morphologically less compact form in solution. It is unclear whether this subtle discrepancy is attributable to crystal packing or due to differences in the buffer conditions employed in these two studies (pH 7.2 for the crystal^22^ and pH 8.0 for cryo-EM^29^). We were thus prompted to investigate potential GNNV structural changes under different pH conditions with cryo-EM (Table S1). We immersed native GNNV virions or respective VLPs in buffers of different pH values corresponding to the weakly alkaline condition (8.0), weakly acidic condition (6.5), and acidic condition in late endosomes (5.0). Cryo-EM images (Figure S1) revealed a spikier appearance of the GNNV VLPs at pH 8.0 and 6.5 than at pH 5.0. By using three-dimensional (3D) reconstruction with icosahedral symmetry, we determined cryo-EM structures of GNNV VLPs at pH 8.0, 6.5, and 5.0 (Figure 1), as well as virions at pH 6.5 and 5.0 (Figure S2), with overall resolutions in the range of 2.82–4.36 Å (Figures S3, S4 and Table S1). These results indicate that lowering the pH prompted GNNV particles to undergo gross structural alteration (Figures 1 and S2), with VLP and virion structures at the same pH being virtually indistinguishable (Figure S2), affirming the use of VLPs as surrogates of virions for vaccine development.^33^

Notably, the diameter of the GNNV particle under acidic conditions (pH 5.0) was smaller (370 Å) than under weakly alkaline (pH 8.0) or neutral (pH 6.5) conditions (380 Å) (Figure 1A). This reduced size is due to an ≈5 Å displacement of the protrusions toward the capsid shell (Figure 1B), with radial density and cross-sectional analyses showing that the shell size remained virtually unchanged (Figures S5 and S6). Moreover, the shell morphology was relatively unperturbed by the acidic conditions (Figure 1C). Closer examination of the protrusions revealed that not only were they positioned closer to the shell in the acidic pH but they also became more compact and were rotated clockwise by ∼20° (Figure 1B, Movies S1 and S2). This repositioning prompted our hypothesis that the linker between the P-domain and S-domain is malleable (Movie S3), prompting further investigation of the detailed mechanism (Figures S3 and S4). Thus, our cryo-EM imaging has revealed that GNNV structures are pH-responsive for the first time, with protrusions on GNNV adopting an erect position under weakly alkaline and neutral conditions but a prone position under acidic conditions.

### NMR Spectra Indicate That the P-Domain of GNNV Contains Highly pH-Sensitive Regions

To investigate the behavior in solution of the protrusions as isolated entities instead of on the GNNV capsid, we cloned and expressed the P-domain (aa 221–338) plus the flexible linker (aa 214–220, linking the P-domain and S-domain) from GNNV (GNNV-P) for structural analysis using NMR spectroscopy and sedimentation velocity analytical ultracentrifugation (AUC). GNNV-P had a molecular weight of 13.8 kDa. Notably, neither the N-terminal linker (aa 214–219) nor the C-terminal region (aa 337–338) were observed in a previously generated X-ray structure of GNNV-P (PDB 4RFU), likely due to their high flexibility.^22^ In the buffer we used for solution analysis, we excluded divalent ions or small molecules used to induce the crystal formation.^22^

**Figure 2:**
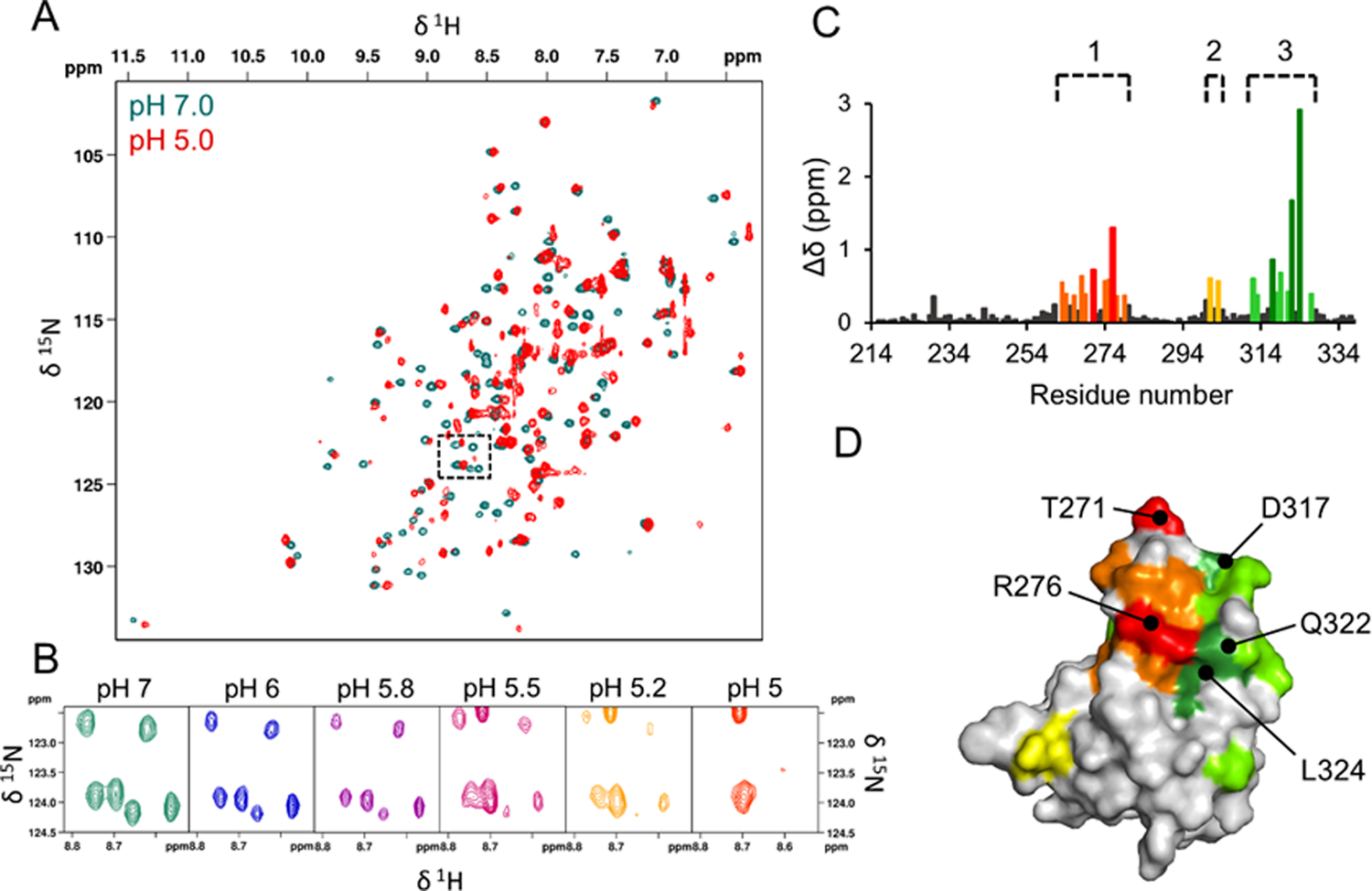
Effects of pH on GNNV-P in solution. (A) ^1^H–^15^N HSQC spectra of GNNV-P recorded at pH 7.0 (blue) and 5.0 (red). (B) Small box in panel (A) is enlarged. The six peaks, in green, at pH 7.0 are from Y279, L263, I222, I323, Q322, and R321, and the peaks, in red, at pH 5.0 are from E217 and I222. Y279, L263, I323, and Q322 disappear in the ^1^H–^15^N HSQC as the pH decreases. (C) Residue chemical shift perturbations (CSPs) by comparing ^15^N-labeled (pH 7.0) and ^13^C-, ^15^N-, and D-labeled (pH 5.0) HSQC spectra. The pH-sensitive Regions I (L263–Y279), II (W301–N303), and III (V312–V327) are highlighted in orange, yellow, and green, respectively. Residues with CSPs >2 standard deviations in pH-sensitive Regions I and III are highlighted in red and dark green, respectively. (D) pH-sensitive residues presented in panel (C) are mapped onto a surface representation of the GNNV-P crystal structure,^22^ with the same color scheme as in panel (B).

We previously collected two-dimensional (2D) ^1^H–^15^N heteronuclear single quantum coherence (HSQC) spectra for GNNV-P at pH 7.0.^34^ To explore the pH dependence of GNNV-P, we conducted a pH titration experiment to cover a wide range of pH values (7.0, 6.0, 5.8, 5.5, 5.2, and 5.0; see Figure 2A for pH 7.0 and 5.0). Our pH titration experiment revealed signatures of pH-sensitive residues, the peaks of which disappeared as the pH decreased (Figure 2B). Moreover, we observed a significant line broadening with the reduction of SNR for the ^1^H–^15^N HSQC spectra at pH 5.0 (Figure 2A), indicating that GNNV-P may undergo a low-pH-induced conformational change or oligomerization, where the latter would induce issues of molecular tumbling and spin relaxation to degrade the spectral quality. To test if this pH-dependent property is reversible, we monitored the 2D HSQC spectra by titrating at increasing pH from 5.0 to 7.0, which showed that the pattern for each pH value could be reproduced, indicating that the pH-dependent changes are reversible (data not shown).

To improve the quality of the pH 5.0 spectra, we utilized deuterium (D) labeling since replacing the ^1^H nuclei with D (^2^H) can suppress strong ^1^H–^1^H dipolar interactions to mitigate spin diffusion. The resulting uniformly ^13^C-, ^15^N-, and D-labeled GNNV-P yielded a well-resolved 2D HSQC pattern at pH 5.0 (Figure S7) as those peaks missing in the ^1^H–^15^N HSQC spectra at pH 5.0 reappeared, allowing us to assign backbone resonances for further chemical shift perturbations (CSPs) analysis. We identified 98.3% of the expected amide ^1^HN and ^15^NH resonances (115 out of 117 nonproline residues), with the exception of residues T214 and L325 (both of which precede prolines in the sequence). Moreover, backbone ^13^Cα, ^13^Cβ, and ^13^C′ resonances were assigned with 98.2–98.4% completeness (see Notes for Data Availability information).

Chemical shift perturbations (CSPs) reflect changes in the chemical environment of the atomic nuclei. These changes can arise from protein interactions or conformational changes. Mapping residues displaying CSPs onto a protein structure can help identify interaction sites.^35^ In principle, CSPs can be analyzed for each residue at each pH titration point (Figure 2B), but we opted to analyze GNNV-P CSPs by comparing the chemical shifts recorded in the ^15^N-labeled HSQC spectra at pH 7.0 and those in the deuterium (D)-labeled HSQC spectra at pH 5.0 (Figures S7), representing the initial and final points of our pH titration experiment (see Figure S8). With nearly all of the chemical shifts assigned for the ^1^HN and ^15^NH data at pH 7.0^34^ and 5.0 (Figure S8), we applied CSPs analysis^36^ to identify residues displaying significantly perturbed chemical shifts relative to pH 7.0, i.e., CSPs greater than one standard deviation from the mean. In Figure 2C, we show the backbone CSPs per residue based on amide nitrogen and proton chemical shifts. Intriguingly, residues displaying pronounced CSPs are clustered into three regions: Region I (L263–Y279), Region II (W301–N303), and Region III (V312–V327). Regions I and III are spatially proximal to each other, as determined by mapping the pH-sensitive residues onto the GNNV-P crystal structure^22^ (Figure 2D). Since residues with pronounced CSPs are likely involved in protein interactions and/or conformational changes,^35^ we speculate that these three regions undergo low-pH-induced interactions or conformational changes. Notably, these regions in GNNV-P that confer pH sensitivity coincide with three β-strands in the crystal structure.^22^

### Determination of the Structure of GNNV-P in Solution at pH 7.0 Reveals That aa 311–330 Form a Long Flexible Loop

**Figure 3:**
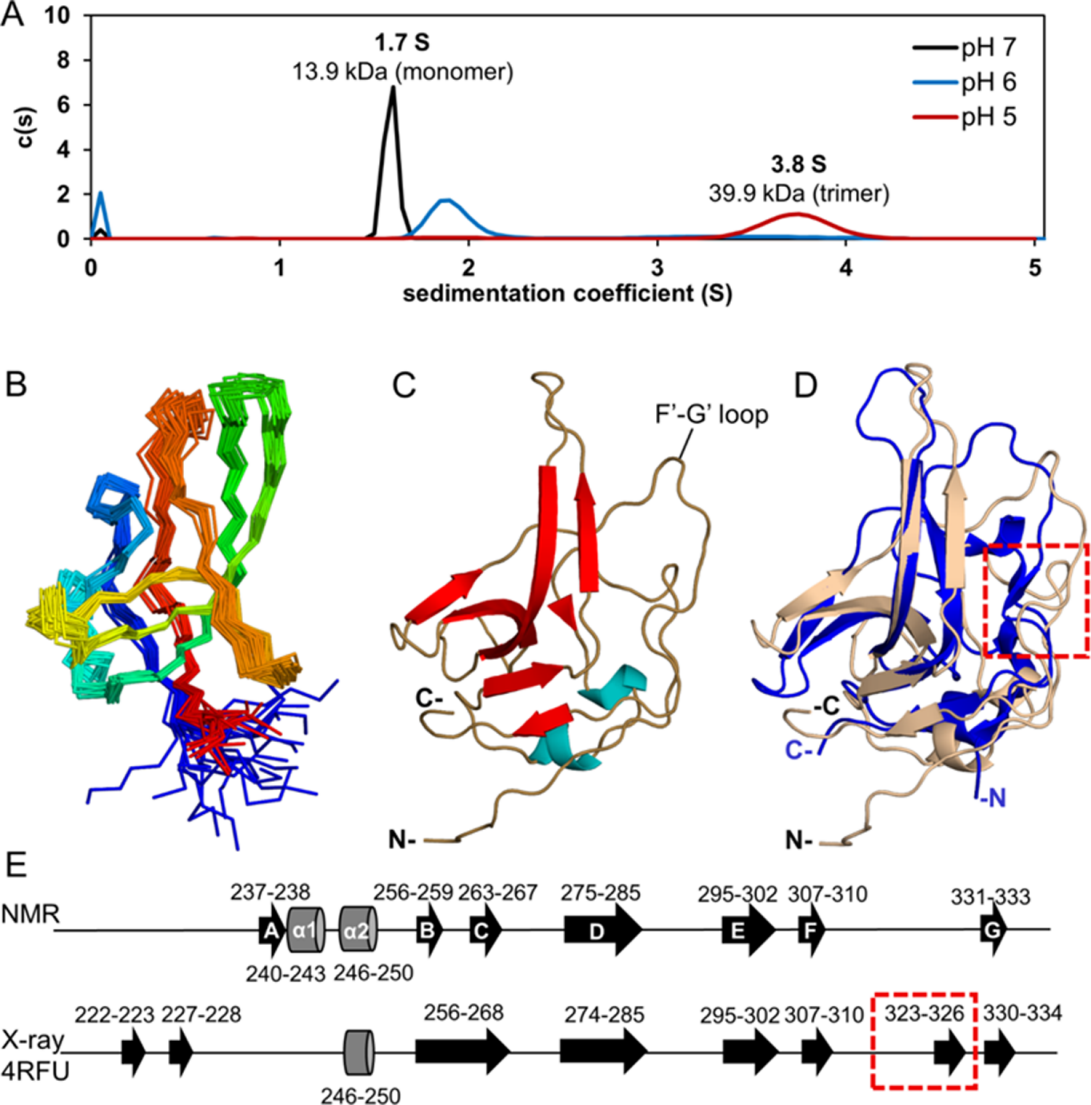
Structure of GNNV-P determined in solution at neutral pH. (A) Sedimentation velocity analytical ultracentrifugation (SV AUC) data of GNNV-P obtained at pH 7.0 (black), 6.0 (blue), and 5.0 (red) fitted to a continuous sedimentation coefficient distribution c(s) model. Sedimentation coefficients and the molecular weight determined by SEDFIT are denoted above each peak. (B) Backbone (N, Cα, and C′) superimposition of the ensemble of 20 low-energy conformations. The N-terminal is colored blue, and the C-terminal is colored red. (C) Cartoon representation of the GNNV-P solution structure with β-strands and α-helices highlighted in red and blue, respectively. (D) Superimposition of the GNNV-P structure determined by NMR at neutral pH (pink) and by the X-ray crystal (PDB 4RFU, blue). (E) Schematic representation of GNNV-P secondary structures, as determined by NMR (pH 7.0) and X-ray crystallography (pH 6.5). β-strands are displayed as black arrows and α-helices as gray barrels, with bordering residues indicated.

Based on the X-ray crystal structure determined under neutral conditions,^22^ GNNV-P was assumed to form a trimer in solution at pH 7.0. Surprisingly, our AUC experiments showed that GNNV-P molecules predominantly adopted a monomeric form at pH 7.0 and assumed only the trimeric form in an acidic condition (pH 5.0) (Figures 3A and S9). Accordingly, we set out to determine the solution structure of GNNV-P based on the HSQC spectra at pH 7.0 (Figure 2A), which exhibited well-dispersed cross-peaks with sharp resonances, indicative of a well-folded GNNV-P structure in solution.

From the ^1^H, ^13^C, and ^15^N backbone and side-chain chemical shift assignments for 122 GNNV-P residues (except for Thr214, Leu228, and Gly270) at pH 7.0,^34^ we calculated the solution structure of GNNV-P using nuclear Overhauser effect (NOE)-derived ^1^H–^1^H distance restraints, chemical shift-derived dihedral angle restraints, and hydrogen bonds inferred from hydrogen–deuterium exchange (Figure S10) (see the Supporting Information for NMR structural determination details). The respective calculation statistics are summarized in Table S2.

In Figure 3B, we present an ensemble of 20 low-energy solution structures, with a representative structure illustrated in Figure 3C. This solution structure of GNNV-P is cone-shaped, similar to the crystal structure (PDB entry 4RFU; see superimposed structures in Figure 3D). The folded GNNV-P tertiary structure comprises a number of secondary structures including antiparallel β-strands and α-helixes, with the order βA-α1-α2-βB-βC-βD-βE-βF-βG (Figure 3E). However, close comparison of the solution and crystal structures revealed a subtle difference; i.e., GNNV-P in solution exhibits a long flexible F′–G′ loop for aa 311–330 (Figure 3E), but this loop is interrupted by a short β-strand (aa 323–326) in the crystal structure (Figure 3E). Interestingly, this loop lies in the trimeric interface of the crystal structure (Figure S11).

### MD Simulations Predict That GNNV-P Forms a Trimer at pH 5.0 with Critical Subunit Interactions Perturbed by Site-Directed Mutagenesis

**Figure 4:**
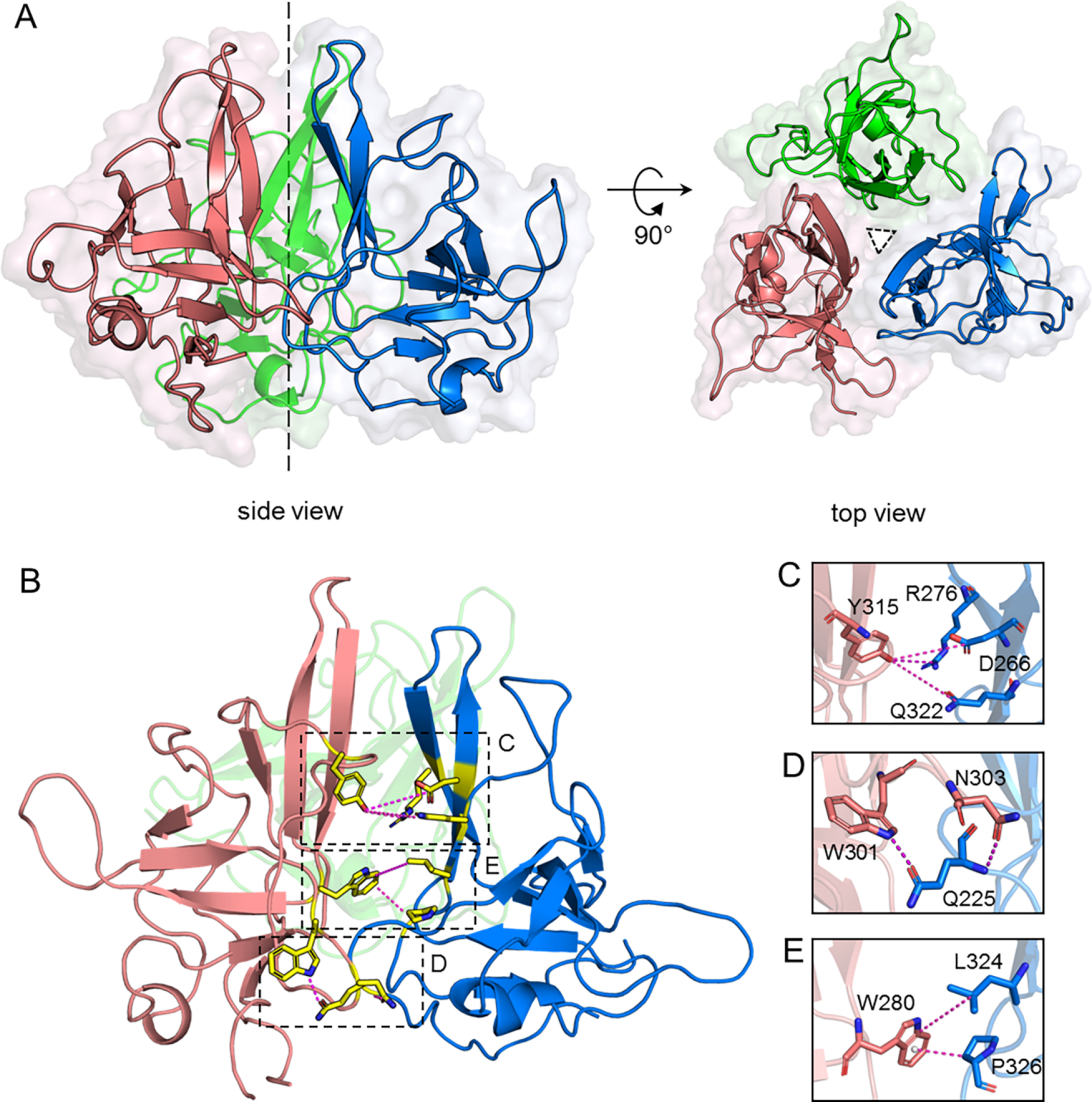
MD-predicted structure of the GNNV-P oligomer at low pH. (A) Top and side view cartoon representations of the GNNV-P trimer formed under acidic pH conditions, as determined by molecular dynamics (MD) simulations. Chain A (blue), chain B (pink), and chain C (green). (B) Intermolecular interactions between neighboring GNNV-P units in the trimer. Residues at the trimeric interface are depicted as yellow sticks, and interactions are shown as dashed magenta lines. (C–E) Details of trimeric interface interactions between GNNV-P chains A and B, as boxed in panel (B).

Our AUC experiments clearly showed that GNNV-P molecules adopt a monomer at pH 7.0 and a trimer at pH 5.0 (Figure 3A). Note that the molecular weight for the pH 6.0 profile (Figure 3A) could not be readily assigned as it likely represents species of heterogeneity stemming from monomer–oligomer exchange (see Figure S9). Identification of subunit interactions for stabilizing the trimer using traditional NMR structural determination is faced with challenges for homooligomers. Thus, we employed an *in silico* approach to the subunit interactions in the GNNV-P trimer. We used molecular dynamics (MD) simulations, a well-established method for understanding protein dynamics, to track GNNV-P structural transitions from the monomer to the trimer. To perform this experiment, we utilized our GNNV-P solution structure determined at pH 7.0 and adjusted the protonation state of amino acid side-chain groups to pH 5.0. Three separate GNNV-P molecules without any symmetry imposed on their spatial arrangement were used as an initial point for our MD simulations, which resulted in a stable trimer (Figure 4A,B), as evidenced by the overall root mean square deviation (RMSD) quickly reaching a stationary phase along the MD time trajectory (Figure S12).

In this complex, the three GNNV-P molecules tightly associated with one another via identical interfaces between neighboring A/C, A/B, and B/C subunits, with the total buried interfacial area estimated by PDBePISA as 1560 Å^2^.^37^ Moreover, 3-fold rotational symmetry emerged for the arrangement of the three GNNV-P molecules, largely matching the structural arrangement of GNNV molecules in the crystal model (Figure S13).^22^ In addition to predicting trimer formation, our MD simulations revealed specific interactions at the inter-GNNV-P level that could be readily verified against the NMR experimental data or tested by using mutagenesis.

Therefore, we searched the MD-predicted structure for residues in the trimer interface, guided by the pH-sensitive regions we had already identified (Figure 2C,D). We detected three pairs of contacts between the side-chains of one subunit and those of a neighboring subunit: Y315 with D266/R276/Q322 (Figure 4C); Q225 with W301/N303 (Figure 4D); and W280 with L324/P326 (Figure 4E). Notably, some of these contacts are disfavored at neutral pH as they are sterically hindered by the F′–G′ loop. Among the three interacting pairs, W280 with the L324/P326 pair seems to be relatively strong as the interactions are dominated by hydrophobic and CH–π interactions, whereas the other two pairs represent either polar interactions or primarily hydrogen bonds. To establish if these three MD-predicted inter-GNNV-P interactions are plausible, we generated three sets of single alanine mutants and assayed their impacts on GNNV-P oligomerization by means of AUC. The first set of mutations encompassed L324A, P326A, W280A, and I323A. As shown in Figure S14, the L324A, P326A, or W280A mutations abolished oligomer formation at low pH (5.0), indicating that this MD-predicted pair of interactions is indeed critical to stabilizing the trimer. As a control, we also mutated I323, a residue in the same pH-sensitive region, but that does not exhibit intermolecular interactions in the MD-predicted structure nor exhibited significant CSPs. As expected, the I323A mutation did not interfere with the formation of GNNV-P oligomers at low pH (5.0). AUC data for the second set of mutations showed that Q322A impaired oligomerization at pH 5.0, whereas R276A still permitted trimer formation, implying that residue Q322 is critical, whereas R276 is not. Regrettably, we were not able to perform AUC experiments for the third set of mutations as the W301A mutant protein failed to fold correctly. Nevertheless, our mutagenesis study verified the importance of the inter-GNNV-P interactions predicted by MD simulations, validating the MD simulation results. We also noticed a conserved histidine at position 281 of GNNV-P (Figure S15) and postulated that it might serve as a histidine switch through pH-induced protonation/deprotonation, as utilized for enveloped virus activation under acidic conditions.^38^ However, the AUC of a H281Y mutant ruled out that this histidine is involved in low-pH-induced GNNV-P oligomerization (Figure S14). The overview of GNNV-P mutants and their effect on GNNV-P low-pH-induced oligomerization is summarized in Table 1.

**Table 1:** List of GNNV-P Mutants

residue	intermolecular interactions at pH 5.0	mutation	AUC results (pH 5.0)
Region I			
R276	H-bond (Y315)	R276A	oligomer
W280	CH–π (P326)	W280A	monomer
	C–π (P326)		
	hydrophobic (L324)		
H281		H281Y	oligomer
Region II			
W301^a^	H-bond (Q225)	W301A	N/A
Region III			
Q322	H-bond (Y315)	Q322A	monomer
	hydrophobic (I300)		
I323		I323A	oligomer
L324	hydrophobic (W280)	L324A	monomer
P326	CH–π (W280)	P326A	monomer
	C–π (W280)		

a Mutation resulted in a misfolded protein.

### NMR Signatures of GNNV-P Conformational Change at Low pH

**Figure 5:**
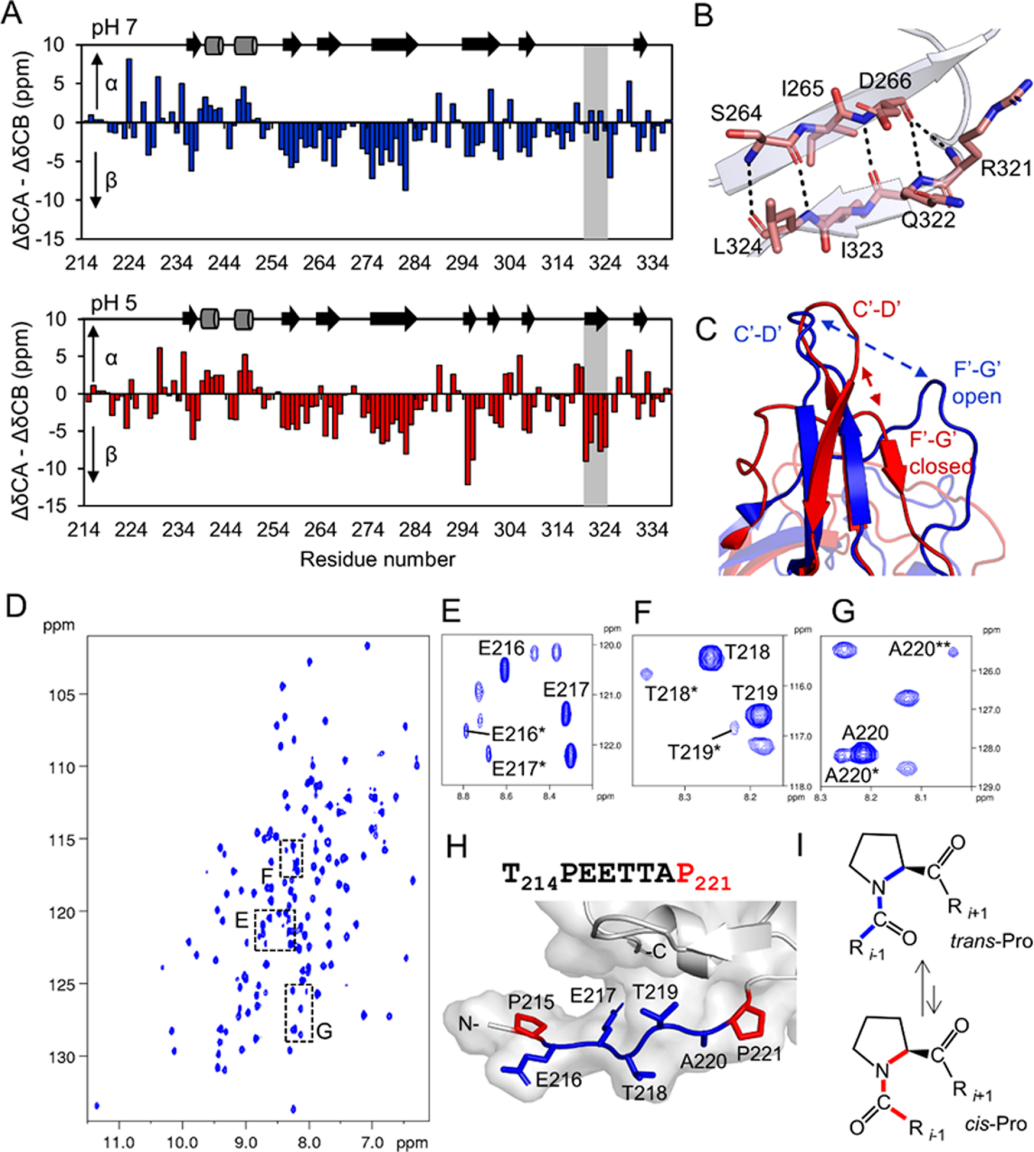
GNNV-P undergoes a low-pH-induced conformational change. (A) Secondary structure propensities of GNNV-P at pH 7.0 and 5.0, calculated using secondary chemical ΔCα–ΔCβ shifts and plotted against the amino acid sequence. The Q320–P324 region showing an increased β-strand propensity at pH 5.0 is highlighted in gray. The secondary structures are shown above each chart, with arrows and cylinders representing β-strands and α-helices, respectively. (B) MD simulations revealed the formation of a short β-strand (comprising residues Q322–L324) within the F′–G′ loop at low pH. Hydrogen bonds between R321–L324 and D266–S264 were identified as stabilizing this region. (C) Superimposition of GNNV-P at pH 7.0 (NMR structure, blue) and 5.0 (MD model, red), showing the open and closed conformations of the F′–G′ loop, respectively. (D) ^1^H–^15^N HSQC spectra of uniform U–D-, ^13^C-, and ^15^N-labeled GNNV-P at pH 5.0. The regions with duplicate signals for residues 216–220 in the linker region are marked with black boxes. (E–G) Details of the uniform U–D-, ^13^C-, and ^15^N-labeled GNNV-P ^1^H–^15^N HSQC spectra highlighted by black boxes in panel (D). (H) Sequence of the N-terminal linker T214-P221 and a stick representation of the linker region with proline residues colored red and residues presenting duplicate NMR signals colored blue. (I) Schematic representation of *cis–trans* isomerization of an Xaa–Pro peptide bond. Proline residues can switch between the *trans* (blue) and *cis* (red) conformations.

Apart from capturing GNNV-P trimerization under acidic conditions, our MD simulations indicated a change in the GNNV-P secondary structure at pH 5.0. This conformational change can also be verified against the NMR experimental data. Compared to backbone CSPs, Cα and Cβ CSPs are more sensitive to a secondary structure change. In searching for residues with pronounced Cα and Cβ CSPs induced by low pH (Figure 5A), we identified a subregion within Region III (aa 312–327) (Figure 5A), exhibiting an increased β-strand propensity at pH 5.0. This subregion is composed of residues P320-L324, and it coincides precisely with an MD-predicted region that converted to a β-strand at pH 5.0. This region in the MD-predicted structure formed intramolecular hydrogen bonds with residues S264 and D266 (Figure 5B), two residues that belong to β-strand C (aa 263–267) (Figure 3E). The impact of these contacts is reflected by the pronounced CSPs of Region I (amino acids 263 and 279) (Figure 3A). Thus, details from our MD simulations could be further validated by CSP data extracted from NMR experiments. Of note, the β-strand of P320-L324 is stabilized by its association with β-strand C; these two strands together with β-strand D form a three-stranded antiparallel β-sheet in the GNNV-P structure at pH 5.0 obtained by MD simulations.

Given the aforementioned conformational change in the F′–G′ loop at low pH (Figure 3E), the space between the F′–G′ and C′–D′ loops collapsed (Figure 5C). Consequently, a pocket in GNNV-P open at neutral pH becomes closed at acidic pH, rendering individual GNNV-P units more compact, as evidenced by a decrease in the surface area from 14053 Å^2^ (pH 7.0) to 12160 Å^2^ (pH 5.0) calculated using Pymol.^39^ Since the β-strand of P320–L324 is situated in the center of the trimer (Figure S13), its switching from the loop configuration mitigates inter-GNNV-P steric hindrances that, together with neutralization of the interfacial repulsing electrostatic potential at pH 5.0 (Figure S16), results in a compact trimer. This detailed mechanism nicely explains the low-pH-induced compaction of protrusions on GNNV particles observed by cryo-EM (Figure 1A,B).

To understand the linker malleability proposed by our cryo-EM observations, we searched the NMR data for residues in the linker region (214–220 aa). During our assignments of NMR spectra for GNNV-P at pH 5.0, we observed duplicate NMR signals (Figure 5D–G) for residues preceding the proline residue (P221) situated at the junction between the linker and the P-domain. This result indicates that the linker gradually shifts among different conformations, possibly due to the peptide bond of P221 undergoing *cis*-*trans* isomerization at pH 5.0 (Figure 5H,I). Our MD simulations support this conformational change of the linker around P221 (Figure S17). However, due to peptidyl bond isomerization being a slow process, directly capturing the switching from a *trans* to *cis* conformation was not possible with our MD simulations. Although the *trans* configuration is favored for peptide bonds, interconversion between the *cis* and *trans* configurations for a Xaa–Pro peptide bond has been previously demonstrated.^40^ In addition, an increased population of P221 in *cis* conformation at low pH has also been inferred by analyzing our ^13^C chemical shifts data using the PROMEGA Web server.^41^ An additional NMR signature for the malleability of the linker is evidenced by the signal for residue A220 in 2D HSQC spectra at pH 5.0, which exhibited various Cα and Cβ chemical shifts in the HNCACB spectra (Figure S18). Taken together, our NMR results provide spectral evidence to support that the linker between the P-domain and S-domain of GNNV is malleable.

### Interactions of GNNV-P with Host Surface Glycan Receptors

Upon encountering a host cell, the protrusions of GNNV-P represent an immediate viral structural motif for the virus to engage with receptors on the host cell surface. Cell surface glycans such as sialic acids have been reported as common cell receptors for distinct NNVs.^42^ By using molecular docking analysis, Nishizawa et al. identified interactions between the terminal moiety of sialic acid and a GNNV protrusion.^28^ That analysis was based on the then available three-dimensional structure of the GNNV P-domain as a compact trimer obtained from X-ray crystallography.^22^ However, our NMR solution structure indicates that the GNNV P-domain adopts a monomeric form under the pH conditions when GNNV engages with host cell surfaces.

**Figure 6:**
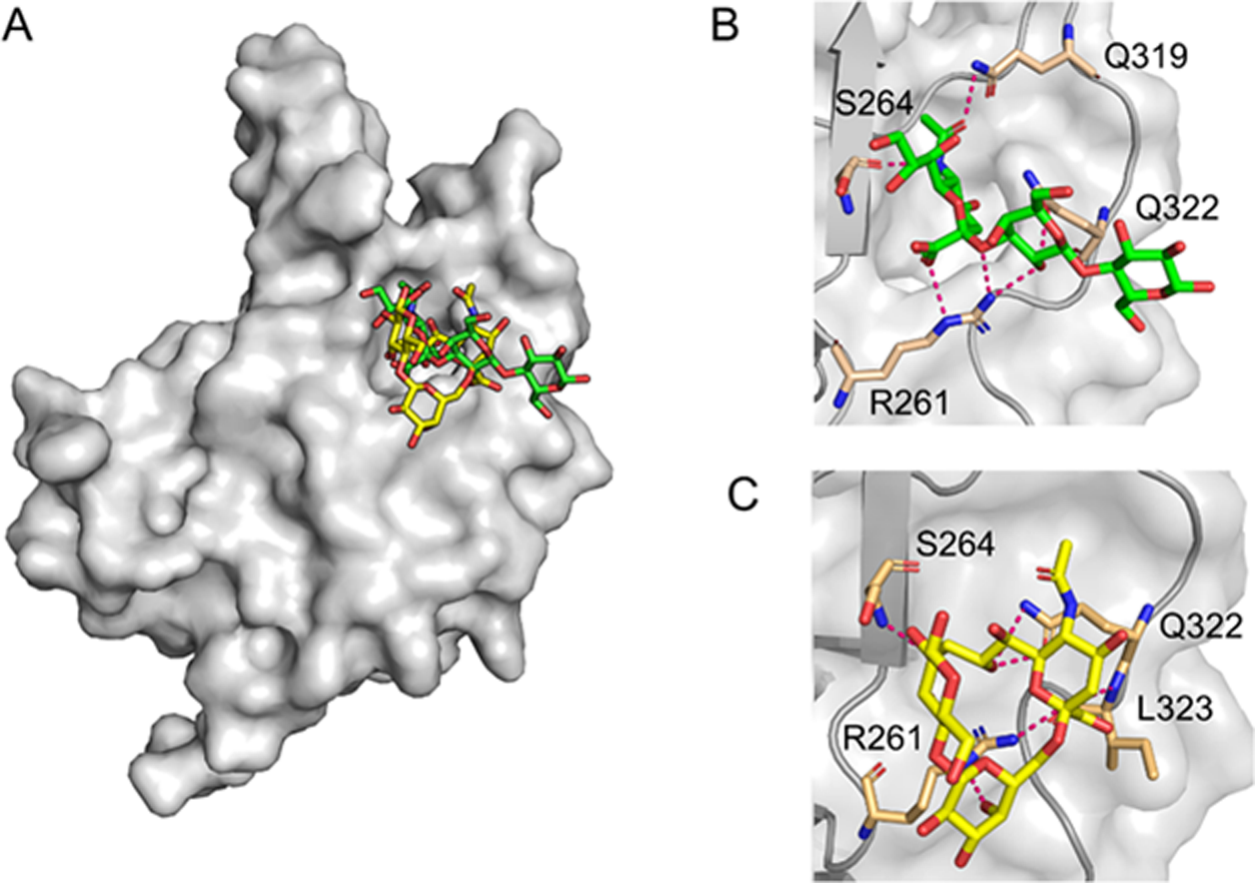
Model of Neu5Ac-Lac binding to GNNV-P at neutral pH. (A) Surface representation of the GNNV-P monomer in complex with Neu5Ac-(α2,3)-Lac (green) and Neu5Ac-(α2,3)-Lac (yellow). (B) Details of Neu5Ac-(α2,3)-Lac binding to GNNV-P in the open pocket conformation. (C) Details of Neu5Ac-(α2,6)-Lac binding to GNNV-P in the open pocket conformation. In panels (B) and (C), residues interacting with Neu5Ac-Lac are shown as sticks, and selected contacts between GNNV-P and Neu5Ac-Lac are represented by pink dashed lines.

Thus, using our P-domain structure at pH 7.0, we could reevaluate potential modes of interaction between sialic acids and GNNV-P. To do so, we conducted a molecular docking analysis with our solution structure of GNNV-P (pH 7.0) against two sialoside isomers, i.e., Neu5Ac-(α2,3)-Lac and Neu5Ac-(α2,6)-Lac. Remarkably, as shown in Figure 6A, the terminal Neu5Ac of both ligands could be inserted deep into the pocket formed by the F′–G′ loop (Figure 5C). The *N*-acetyl chain of Neu5AC was located inside the binding pocket, and the *N*-acetyl methyl group was oriented toward the hydrophobic part of this pocket (Figure 6B,C), with Neu5Ac binding being further stabilized by a number of interactions with the conserved residues lining the pocket. Those contacts include hydrogen bonds between R261 and the sialic acid carboxylate and hydroxyl groups of the penultimate galactose. The *N*-acetyl group of Neu5Ac-(α2,3)-Lac was stabilized by hydrogen bonds with S264 and Q319, and another hydrogen bond formed between the hydroxyl group of the penultimate galactose and Q322 (Figure 6B). For Neu5Ac-(α2,6)-Lac, sialic acid formed additional interactions with S264, Q322, and I323 (Figure 6C).

Together, these specific interactions endow a strong binding affinity between GNNV-P and Neu5Ac-(α2,3)-Lac or Neu5Ac-(α2,6)-Lac, with free energies of −6.42 and −6.48 kcal/mol, respectively. Significantly, those interactions could be augmented multivalently by the three GNNV P-domains that form a viral capsid protrusion. As a control, we also performed a molecular docking analysis using our trimeric structure (pH 5.0) against the same two sialoside isomers. Since the pockets in the trimeric structure are closed at this pH due to a conformational switching in the F′–G′ loop, Neu5Ac-(α2,3)-Lac or Neu5Ac-(α2,6)-Lac could no longer insert into the pocket. Instead, they attached to a cavity at the apex of the GNNV-P trimer (Figure S19), in agreement with the docking results of Nishizawa et al.^28^ It is noticed that this cavity was referred to as a “pocket” by Nishizawa et al.,^28^ which differs from the pocket uncovered herein.

## Discussion

Here, we provide the first glimpse of the structural changes of GNNV particles and reveal that the changes are programmable by external pH conditions. The drastic changes reside with the protrusion, and they are of physiological relevance as the sampled pH values mimic the stages in the journey of virus entry via the endocytic pathway. We next report the first solution structure of the GNNV P-domain (GNNV-P), in which an unexpected feature of structural nuance in the GNNV P-domain is revealed: a long and continuous flexible loop, the F′–G′ loop, outlining a previously unknown deep pocket. By means of MD simulations, we identify a region in this loop that can undergo a disorder-to-order transition triggered by an acidic pH, which, in turn, elicits P-domains to form a compact trimer. There are a number of significant outcomes from this work. First, this work shows that GNNV particles are dynamic biological nanomaterials, and challenge the notion that the crystal structure represents “the” form for host cell docking.^22^ Second, since the pH condition in which each GNNV structure was investigated corresponds to a defined stage along the route of entry, our structural findings represent high-resolution snapshots in a video that describes NNV configurations along the journey of virus entry, which can be obtained by live imaging techniques but with much poorer resolution. Third, our results represent a rare study that connects the structural transitions observed at the coarse level—the loose protrusion configuration on the GNNV capsid at near-neutral conditions (pH 6.5–8.0) to the compact configuration at the acidic condition (pH 5.0), with those at the atomic level—the GNNV-P monomer structure at a neutral condition (pH 7.0) and the acid-induced GNNV-P trimer structure (pH 5.0). Lastly and most importantly, this study may lead to identification of putative druggable sites on NNV.

### Advancement from the Crystal Structure—GNNV Structure Configuration for Cell Attachment

Regarding virus entry, docking of the virion on a host cell surface represents the first step during the journey. GNNVs dock on their host cells, e.g., Grouper fin cells, under weakly alkaline conditions as wild Groupers live in relatively warm oceanic regions that exhibit weakly alkaline conditions (pH 8.1) or under neutral conditions (pH 7.0–7.5) for farmed Grouper. A GNNV that has succeeded in entering a host cell is being trafficked through the endosomal system; it would encounter a wide range of pH variations (from pH 8.1 during cell attachment to pH 5.0 in late endosome). Prior to the present study, it has been accepted that the structure utilized for host cell docking is akin to the crystal structure^22^ since it was obtained at a neutral condition (pH 7.2). In this crystal structure, GNNV protrusions adopt the resting/prone and compact configuration, and this configuration is thus believed to be the form interacting with the host cell receptor. Seeing the limitations of using the crystal approach on sampling aqueous conditions, we used cryo-EM to explore GNNV structures in various pH conditions as this method is effective for high-resolution structure determination of noncrystal specimens in a wide range of aqueous conditions. By mimicking those conditions encountered en route of host cell entry via the endocytic pathway, we discovered that the protrusions on the GNNV capsid adopts an erect/rising position with loose configuration at the docking condition (pH 8.0) and early endosome condition (pH 6.5), but compact and rest on the capsid shell at the late endosome condition (pH 5.0). Structural comparison shows that the crystal structure of GNNV^22^ resembles the cryo-EM structure at pH 5.0, suggesting that the crystal structure probably best represents an intermediate structure that a GNNV particle adopts in the late stages of virus entry, but less likely to be the form of GNNV interacting with host cell receptors as has been implicated.^28^

### Low-pH-Induced P-Domain Structural Transitions and Self-Assembly

As our cryo-EM analysis could not resolve the densities in the protrusion region of the GNNV virion (Figures S3 and S4), this analysis failed to offer a detailed mechanism to explain the GNNV protrusion dynamics. We therefore expressed the P-domain alone (GNNV-P) in order to obtain its atomic structure by solution NMR. The analytical ultracentrifugation results surprisingly show this GNNV-P protein is largely monomeric in solution at neutral pH but assembles into a trimer at acidic pH. We then determined the NMR structure for this monomer to yield the first atomic structure of the P-domain (pH 7.0), revealing that a region of 20 amino acids (aa 311–330) in the P-domain adopts a long flexible loop, contrasting to it being interrupted by a short β-strand (aa 323–326).^22^ By using MD simulations, we showed that this long flexible loop could undergo unusual pH-dependent secondary structure changes. At pH 7.0 this F′–G′ loop seems to be disordered, but at pH 5.0 a subregion in it acquires a β-strand (aa 320–324). It is noted that the subregion of β-strand is not exactly the same as the counterpart in the crystal structure.^22^ This structural transition enables this F′–G′ loop to switch from a continuous loop to an interrupted loop. Such conformational change at the level of the secondary structure has a number of significant consequences at the level of tertiary and quaternary structures. First, it causes the pocket outlined by the F′–G′ and C′–D′ loops to close (Figure 5C): at neutral pH, the pocket is open as the F′–G′ and C′–D′ loops lie far apart, so the pocket is open, whereas at acidic pH it becomes closed as the β-strand emerging from the F′–G′ loop forms a three-stranded β-sheet with C′ and D′ β-strands. Second, this conformational switching apparently ameliorates loop-elicited steric hindrance between neighboring P-domains that has prevented the P-domain from self-assembling into a trimer (Figure 4). This trimer is further stabilized by inter-GNNV-P interactions, of which critical contacts between subunits such as P326 and W280 have been verified by site-directed mutagenesis. Notably, our study demonstrates that at acidic conditions, the trimer of P-domains can be stabilized merely by protein–protein interactions in the absence of calcium ions as reported in the crystal structure,^22^ since our study has omitted divalent ions used for crystallization.^22^ This apparent discrepancy is understandable: calcium ions have the power to neutralize the electrostatic repulsion conferred by aspartate acids on the trimer interfaces, but the role of calcium ions is fulfilled by protons in acidic conditions.

### Deep Pocket in the P-Domain and Its Implications

Since the protrusions on the GNNV capsid adopt a loose configuration at neutral aqueous conditions for host cell docking, the NMR structure of the P-domain (monomer) obtained at neutral conditions would represent a more appropriate system relative to the crystal structure (trimer) for evaluating how NNVs interact with host receptors. Strikingly, our molecular docking analysis of the interactions between the GNNV-P monomer and short sialylated oligosaccharides revealed the deep pocket in the P-domain outlined by the F′–G′ loop is the site to interact with the terminal sialic acid moiety. The sialic acid moiety is found on glycoproteins on host cell surfaces. Because the tested sialic acids interact with highly conserved residues deep in the pocket, our results support the notion that sialic acids are used as common cell receptors across all NNV genotypes.^42^ It is envisioned that a small molecule targeting this pocket may inhibit the receptor binding.^43^ It is noted that this pocket in the trimeric structure is no longer available, and the receptors of sialic acids are then withdrawn to the apex of the trimer.

**Figure 7:**
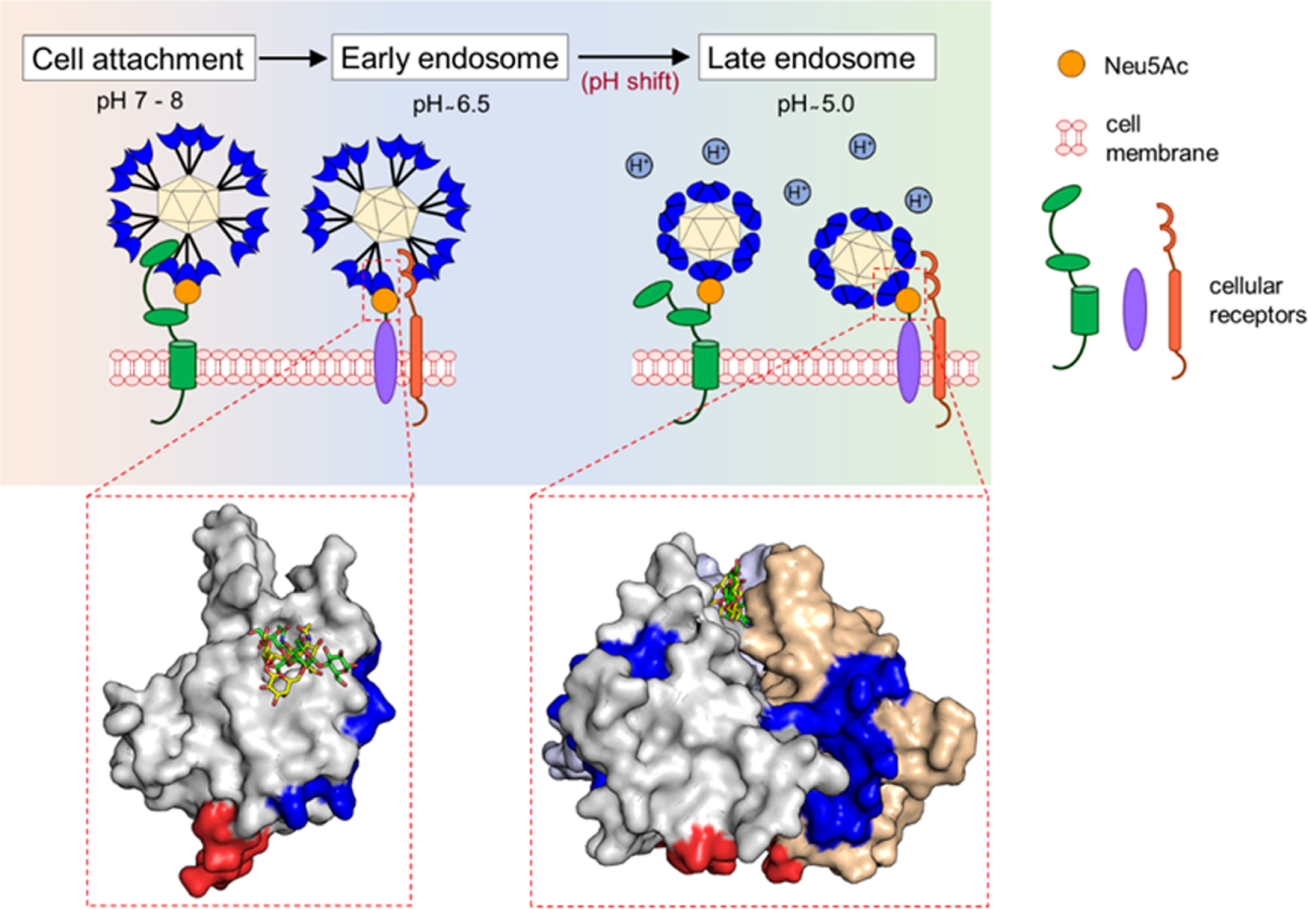
Model of GNNV interactions with host cell surface receptors. During infection, GNNV attaches to cells by interacting with the sialic acid moiety on the host cell surface and with cellular receptors (HSP70, HSP90ab1, or nectin-4). At weakly alkaline or neutral pH for host cell entry, protrusions on the GNNV surface are in extended and loose configuration, with the sialic acid binding pocket open, and linker region (red) and host-determining region (blue) accessible for interactions with cellular receptors. Acidification in late endosomes induces conformational change of the GNNV P-domain to result in resting/prone and compact protrusions. In this configuration, the sialic acid binding pocket is closed so that sialic acids are withdrawn to the tip of the protrusions. In addition, the accessibility of the linker region to a cellular receptor is also reprogrammed, leading to its potential GNNV detachment for endosomal escape. Note that the potential of the protrusion tip in directly interacting with the endosomal membrane may be altered by the pH-induced modification of the protrusion surface hydrophobicity distribution (Figure S20).

Since a common cellular receptor cannot differentiate between NNV types, a protein coreceptor is required to determine the host specificity.^44−46^ Such protein coreceptors include 90ab1^45^ and nectin,^46^ which bind to amino acids 213–230 and 221–238 of the P-domain, respectively. These host-determining regions of NNVs have good overlap with a highly variable sequence (aa 223–244).^47^ As this variable sequence lies close to the linker (aa 214–220), yet distant from the sialic acid binding pocket, we speculate that the pH-associated malleability may play a role in dynamically controlling the accessibility of those host-determining regions. Based on the interactions between the protrusion pocket and sialic acid receptors, as well as those between the protrusion host-determining region and a protein coreceptor, we propose a pH-dependent NNV-host engagement model (Figure 7) crucial for virus entry, where its connection to endosomal escape^48^ warrants further study.

### Malleable Linker That Connects the P-Domain to the S-Domain

Compared with X-ray crystallography or cryo-EM, NMR has a superior advantage of detecting signals from highly mobile regions. The ability to detect these signals revealed pH-associated malleability of the linker that connects the P-domain and the S-domain. The NMR spectra of this linker indicated that it could adopt preferred configurations in acidic conditions, which is made possible by the peptide bond of a conserved proline (P221), situated at the junction of the linker and P-domain, preferring the *cis* configuration (Figure 5). This linker malleability explains the pH-dependent positioning of protrusions on GNNV particles, as observed by cryo-EM.

While we were delving into the atomic details of GNNV-P using NMR analysis, Song et al. investigated mouse and human noroviruses by cryo-EM under various pH conditions^49^ in order to resolve the mystery of classifying caliciviruses based on different protrusion positions in capsid structures. In the “rising” type as shown by human norovirus GII.10 and rabbit hemorrhagic disease virus (RHDV), the protrusions are in the rising position, whereas in the “resting” type as shown by human norovirus GI.1, sapovirus, and San Miguel sea lion virus (SMSV), the protrusions rest upon the virus shell. Like betanodavirus, norovirus is a nonenveloped virus exhibiting pronounced surface protrusions, with each norovirus protrusion composed of two copies of the P-domain. Like GNNV, the norovirus P-domain is connected to an S-domain via a flexible hinge.^50^ This pH-dependent cryo-EM investigation of noroviruses^49^ has revealed that norovirus protrusions rest on the virus capsid in neutral pH (<8.0) but become erect at alkaline pH (>8.0). This ability of dynamic switching of protrusion positions is apparently crucial for viral infection as the resting/prone protrusions are adopted in aqueous conditions favoring virus entry and seem more accessible to cellular receptors.^49^ Comparison between GNNV and noroviruses indicates that both pH thresholds for triggering the conformational change and the physiology context are significantly different. For noroviruses, protrusion configuration switching occurs at approximately pH 8.0, while that for GNNV occurs at pH 5.0. For noroviruses, the resting/prone form is adopted at the neutral condition encountered at the host cell surface, whereas for GNNV the same form is adopted at acidic conditions in late endosomes. In other words, the infectious form of GNNV is more likely with rising protrusions, whereas that of the norovirus is with the resting protrusions. Those differences may be ascribed to the different natures of these viruses and the circumstances of the host infection as well. Nevertheless, they may use similar molecular features to respond to environment changes. First, the protrusion domains of both of these viruses are connected to the virus shell by a flexible linker/hinge. Second, the linkers and hinges of both viruses undergo conformational changes. Third, norovirus does bear a number of prolines in its hinge presumably pivotal for its protrusion malleability, e.g., the PPT (Pro–Pro–Thr) sequence.^50^ Our study shows that the distribution of conformations in the GNNV’s linker might be altered by the change in the pH. In the case of the norovirus, the role played by pH in tuning the hinge’s conformation distribution has not been established. Thus, the pH-associated linker’s malleability learned from our study may offer an insight into the malleability of the norovirus linker. We speculate that the similarity in linker malleability between NNV and the norovirus, a phylogenetically distant virus, might have been acquired through convergent evolution.

Taken together, our discovery of GNNV pH-dependent structural changes by cryo-EM followed by combined analysis using NMR, MD simulations, and mutagenesis has unraveled the inner-working of GNNV protrusion dynamics. Specifically, our findings reveal a unique pH-induced conformational switching mechanism^51^ that involves coupling of the local protein fold change and protein oligomerization.^52,53^ This mechanism might be utilized by NNV as a means of host cell entry and perhaps subsequent survival. Considering anti-NNV strategies, conventional approaches focus on vaccine development,^23,54−56^ for which our finding of GNNV conformational changes can be of benefit with regard to the structure-based vaccine design^57^ of new NNV-neutralizing antibody that has the capacity to access conformation-sensitive epitopes. Nevertheless, most fish vaccines are administrated by injection, which is practically labor-intensive and may induce temporary immunosuppression. Thus, alternative anti-NNV strategies other than vaccines are desired. A pocket in a protein structure usually represents an ideal druggable site.^58,59^ Our finding of the common receptor-binding pocket in the GNNV P-domain may suggest a brand new anti-NNV strategy that uses small molecules or short peptides, which can be easily delivered to farmed fish using fish feed. A small molecule that targets this pocket can disrupt interactions between NNV and a common cellular receptor to broadly inhibit the entry of NNV from host cell entry. In addition, since loops^60^ or other protein motifs^61^ critical for protein–protein interactions^62^ are also suitable targets of small molecules, candidate druggable sites may include the F′–G′ loop and other protein motifs on the trimer interfaces. In conclusion, this work provides new structural insights into NNV with the identification of a number of putative druggable sites on the capsid protrusion in this environmentally and economically important virus and suggests that virus–host interactions can be potentially complex even for a simple virus.

## Methods

### Sample Preparation of GNNV and VLPs for Cryo-EM

GNNV and VLPs were purified using a 10–40% (w/w) sucrose density gradient, as previously described.^14,29^ To prepare GNNV and VLPs in different pH conditions, the purified particles were pelleted down by ultracentrifugation at 30,000 rpm for 3.5 h at 4 °C (Beckman Coulter, OptimaTM L-90K Ultracentrifuge, rotor: SW 41 Ti). The particle pellets were then resuspended overnight in 100 μL of TN buffer (50 mM NaCl, 50 mM Tris-HCl, pH 8.0), MES buffer (50 mM NaCl, 50 mM MES, pH 6.5), or acetate buffer (50 mM NaCl, 50 mM sodium acetate, pH 5.0).

To prepare the cryo-EM samples of GNNV and VLPs, approximately 3.5 μL of protein solution was deposited onto a Quantifoil R1.2/1.3 holey carbon grid (Quantifoil Micro Tools GmbH, Jena, Germany) coated with a thin carbon film. The grid was then rapidly plunged into liquid-nitrogen-cooled liquid ethane and stored in liquid nitrogen until imaging. The cryo-EM grids of GNNV at pH 6.5 and 5.0 were prepared using a Vitrobot Mark IV system (Thermo Fisher Scientific, Hillsboro, OR, USA) at 4 °C and 100% humidity, with a blotting time of 3.5 s. For VLPs at pH 8.0, 6.5, and 5.0, the cryo-EM grids were prepared using a Leica EM GP system (Leica Biosystems, Deer Park, IL, USA) with the sensor off option—the cryo-EM grids of VLPs at pH 8.0 and 5.0 were prepared at 15 °C and 80% humidity, with a blotting time of 1.2 s; whereas the cryo-EM grids of VLPs at pH 6.5 were prepared at 22 °C and 95% humidity, with a blotting time of 0.5 s. All subsequent steps were conducted at the liquid-nitrogen temperature to prevent devitrification.

### Cryo-EM Data Acquisition

The cryo-EM grids containing GNNV virions at pH 6.5 and 5.0 were examined using cryo-ARM (JEOL Ltd., Akishima, Tokyo, Japan) at magnifications of 40 000× and 50 000×, respectively, with pixel sizes of 1.36 and 1.09 Å/pixel. Cryo-EM images of GNNV at pH 6.5 and 5.0 were recorded using a K2 camera (Gatan Inc., Pleasanton, CA, USA) in counting mode, with an exposure time of 8 s for 40 frames. The dose rate was approximately 6.5 electrons/Å^2^ per second, resulting in a total accumulated dose of around 52 electrons/Å^2^ (equivalent to approximately 1.3 electrons/Å^2^ per frame).

The cryo-EM grids containing VLPs at pH 6.5 were examined using Technai F20 (FEI, Hillsboro, OR, USA) at a nominal magnification of 29,000×, resulting in a pixel size of 1.24 Å/pixel. Cryo-EM images of the VLPs at pH 6.5 were recorded using a K2 camera (Gatan Inc., Pleasanton, CA, USA) in counting mode, with an exposure time of 10 s for 50 frames. The dose rate was approximately 5 electrons/Å^2^ per second, resulting in a total accumulated dose of around 50 electrons/Å^2^ (equivalent to approximately 1.0 electron/Å^2^ per frame).

The cryo-EM grids containing VLPs at pH 8.0 and 5.0 were examined using JEM-2100F with a high-contrast pole piece (JEOL Ltd., Akishima, Tokyo, Japan) at a magnification of 50,000×, with a pixel size of 1.16 Å/pixel. Cryo-EM images of the VLPs at pH 8.0 and 5.0 were recorded using a DE-20 camera (Direct Electron LP, San Diego, CA, USA) in linear mode, with an exposure time of 1.5 s for 38 frames. The dose rate was approximately 20 electrons/Å^2^ per second, resulting in a total accumulated dose of around 30 electrons/Å^2^ (equivalent to approximately 0.8 electrons/Å^2^ per frame). The parameters for cryo-EM data acquisition are summarized in Table S1.

### Single-Particle Image Processing and 3D Reconstruction

All cryo-EM image stacks underwent motion correction and dose weighting using MotionCor2^63^ with a 5 × 5 patch. The contrast transfer function (CTF) was determined using CTFFIND4^64^ from the motion-corrected and dose-weighted images. Particle picking was conducted in cryoSPARC^5^ using 2D templates generated from a previously determined VLP cryo-EM map.^29^ After particle extraction and removal of bad particles through 2D classification, the remaining particles were used for further *ab initio* reconstruction and heterogeneous refinement with icosahedral symmetry (*I*). A subset of particles from a good 3D class with more particles and better resolution was chosen for homogeneous refinement with icosahedral symmetry (*I*). Overall resolution was assessed using the Fourier Shell Correlation (FSC) = 0.143 criterion, and local resolution was calculated within cryoSPARC.^65^ The final cryo-EM maps achieved overall resolutions of 3.12 Å (GNNV pH 6.5), 4.36 Å (GNNV pH 5.0), 3.23 Å (VLP pH 8.0), 2.82 Å (GNNV pH 6.5), and 3.52 Å (GNNV pH 5.0) (Figures S3, S4 and Table S1). Visualization of the resulting 3D density maps was performed using the UCSF Chimera.^66^ The details of single-particle image reconstructions of GNNV and VLPs can be found in the flowcharts in Figures S21 and S22, and the cryo-EM reconstruction details are summarized in Figures S3 and S4. Additional information regarding cryo-EM reconstruction statistics is available in Table S1.

### Protein Construct and Site-Directed Mutagenesis

The cDNA encoding the Dragon grouper NNV P-domain (aa 214–338, GNNV-P) was tagged with His_6_-yeast SUMO (Smt3) at the N-terminus and was cloned into the pETDuet-1 vector. GNNV-P mutant constructs—namely, R276A, W280A, H281Y, W301A, Q322A, I323A, L324A, and L325A—were created using plasmids harboring the wild-type GNNV-P sequence and a QuickChange Lightning site-directed mutagenesis kit (Agilent Technologies, CA, USA). Mutations were confirmed by PCR sequencing (Genomics Inc., Taiwan). Primers used for mutagenesis were synthesized by Tri-I Biotech (NTC, Taiwan).

### Protein Expression and Purification

Wild-type and mutant GNNV-P proteins were expressed using a transformed *Escherichia coli* BL21 (DE3) strain according to expression and purification protocols reported previously.^34^ Uniform U-[^2^H, ^13^C, ^15^N] triple-labeled proteins for NMR assignments at pH 5.0 were expressed in M9 minimal medium prepared using 100% D_2_O (Sigma-Aldrich) as the solvent (M9 D_2_O medium), with 1 g/L ^15^NH_4_Cl (Sigma-Aldrich) and 2 g/L U-^13^C_6_-Glucose (Cambridge Isotope Laboratories) as the sole nitrogen and carbon sources, respectively, according to a modified expression protocol. In brief, overnight culture of transformed *E. coli* was first transferred to 1 L of LB medium supplemented with 100 μg/mL ampicillin and grown until the OD_600_ reached a value of 1.0. The cells were then collected by centrifugation and excess LB medium was removed before resuspending the cells in M9 D_2_O medium. Protein expression was induced by the addition of IPTG (final concentration of 0.3 mM) dissolved in 100% D_2_O. Then, the cells were grown for an additional 4 h at 37 °C with shaking at 150 rpm, before being harvested by centrifugation at 8000 rpm for 25 min at 4 °C.

### Sedimentation Velocity Analytical Ultracentrifugation (SV AUC)

The sedimentation velocity experiments were performed in a Beckman Coulter ProteomeLab XL-I analytical ultracentrifuge equipped with a 190–800 nm absorbance optical system. The concentration of protein samples used for SV AUC was in the 0.25–0.4 mg/mL range. The SV experiments were carried out at a rotor speed of 60,000 rpm at 20 °C and absorbance was monitored at λ = 280 nm. The protein sample buffer was used as a reference. Sedimentation coefficients were determined using the SEDFIT v16-1c software.^67^ The sedimentation velocity data was fitted into a continuous c(s) distribution model based on solving the Lamm equation by the least-squares technique.^67^ Buffer density (ρ), viscosity (η), and molecule partial specific volume were estimated using SEDNTERP software.^68^

### NMR Spectroscopy and Structure Determination

All NMR experiments were performed at 298 K on Bruker AVANCE 600, 800, and 850 MHz spectrometers equipped with 5 mm triple resonance TXI cryogenic probes, including a shielded Z-gradient. Samples containing 10% D_2_O were loaded into 5 mm Shigemi NMR tubes for NMR experiments.

The sequence-specific backbone resonance assignments at pH 5.0 were achieved using 1.0 mM U-[^2^H, ^13^C, ^15^N]-labeled GNNV-P protein in 20 mM sodium acetate (pH 5.0), 50 mM NaCl, 0.5 mM ethylenediaminetetraacetic acid (EDTA), 0.02% sodium azide, and 90% H_2_O/10% D_2_O. NMR spectra were processed using Bruker Topspin 3.6 and analyzed using NMRviewJ 9.2.0.^69^ The sequence-specific backbone assignments have been determined by independent connectivity analysis of HNCACB, HNCO, and HN(CA)CO experiments. We completed backbone assignments for 123 of 125 residues (98.3%), with the exceptions of Thr214 and Leu325. These resonance assignments have been deposited into the Biological Magnetic Resonance Data bank with accession code 52218.

GNNV-P structural calculations at neutral pH were carried out in XPLOR-NIH software version 3.8^70^ in NMRbox^71^ using experimentally determined distance restraints, hydrogen bonds, and predicted dihedral angles. Backbone and side-chain NMR assignments of the NNV P-domain at neutral pH were reported previously.^34^ NOE distance restraints were derived from an ^15^N-edited NOESY-HSQC spectrum and they were analyzed using NMRviewJ software.^69^ Hydrogen bonds were identified based on hydrogen/deuterium exchange experiments. The backbone dihedral angle restraints Φ and Ψ were predicted from chemical shifts using the TALOS+ Web server.^72^ For the GNNV-P structural determination, 100 structures were generated according to a standard simulated annealing protocol. Twenty structures with the lowest energy were selected for refinement using the implicit solvation potential and effective energy function (EFFx) in XPLOR-NIH.^73^ The 20 structures with the lowest energy and without reported violations were selected for assessment and quality checking using the protein structure validation suite in the wwPDB Validation server.^74^ The final ensemble of 20 structural conformations of GNNV-P has been deposited in the Protein Data Bank (PDB entry 8XID) and the respective structural statistics are summarized in Table S2.

### Amide Hydrogen–Deuterium Exchange Rate (HXD)

Hydrogen–deuterium exchange between the amide NH signal and D_2_O solvent was monitored using ^1^H, ^15^N HSQC spectra. First, an initial ^15^N HSQC spectrum was collected before freezing GNNV-P in liquid nitrogen and lyophilizing it. The progress of amide hydrogen–deuterium exchange was monitored by collecting ^15^N HSQC spectra for GNNV-P samples at pH 7.0 after redissolving them in 100% D_2_O for 4 h. The hydrogen–deuterium exchange rate was calculated by fitting peak intensities into the exponential decay function *I*(*t*) = *I*_0·e^∧^(−*t*/*x*) using NMRviewJ.^69^

### Chemical Shift Perturbation and Secondary Chemical Shifts Calculation

2D ^1^H–^15^N HSQC spectra of GNNV-P at pH 7.0 and 5.0 were used for the chemical shift perturbation analysis. The chemical shift between pH 7.0 and 5.0 was calculated using the equation , where Δδ_H_ and Δδ_N_ are the ^1^H and ^15^N chemical shift changes, respectively. A scaling factor of α = 0.1 was used to account for the larger ^15^N chemical shift.^36^ Secondary structure propensities were predicted from ^13^C_α_ and ^13^C_β_ chemical shifts based on their deviations from random coil values.^75^

### Molecular Dynamics (MD) Simulation

Simulations for GNNV-P trimer formation were performed using the GROMACS package.^76^ Initially, the protein protonation state was adjusted to pH 5.0 using PDB 2PQR software.^77^ To prepare the initial point for the simulation, three protein molecules were initially packed in a ∼300 Å cubic box using the software PACKMOL.^78^ This step was done to ensure that no repulsive interactions would disrupt or cause errors during the simulations. Using a V-rescale thermostat, the overall temperature of the water and protein was kept constant by coupling each group of molecules independently at 300 K. A Parrinello–Rahman barostat was used to separately couple the pressure to 1 atm in every dimension.^79^ The time constants for the temperature and pressure couplings were set to 0.1 and 2 ps, respectively. A time step of 2 fs was applied by using the leapfrog algorithm to integrate the equations of motion for the system. Periodic boundary conditions were set for the whole system. For the Lennard–Jones and the Ewald sum Coulombic interactions, we set a 1 nm cutoff. The Fourier space part of the Ewald splitting was calculated using the particle-mesh-Ewald method by applying cubic spline interpolation and 0.16 nm grid length on the side.^80^ The TIP3P water model was used and the protein parameters were obtained from the AMBERff99SB-ILDN force field.^81,82^ MD simulations were performed for a total scan length of 100 ns.

### Molecular Docking Analysis

Molecular docking analysis on GNNV-P with two sialoside isomers, Neu5Ac-(α2,3)-Lac and Neu5Ac-(α2,6)-Lac, was carried out using the HADDOCK 2.4 Web server and protein–glycan default settings.^83^ The three-dimensional structures of Neu5Ac-(α2,3)-Lac and Neu5Ac-(α2,6)-Lac were extracted from PDB entries 6TLZ and 6TM0, respectively. The binding energy between GNNV-P and the docked sialosides was estimated using the PRODIGY Web server.^84^

### SV-AUC Data Interpretation and Protrusion Hydrophobicity Analysis

Interpretations of pH 6.0 and 5.5 SV-AUC profiles were performed with cautions according to Schuck and Zhao.^85^ Hydrophobicity patches on P-domain (pH 7.0) and protrusion as trimer of P-domains (pH 5.0) were analyzed using Kyte–Dolittle analysis.^86^

## Data Availability

Cryo-EM maps of GNNV VLP at pH 8.0, 6.5, and 5.0 are available from the EMDB data bank with accession numbers EMDB-39212, 39213, and 39214, and the corresponding atomic models are with PDB entries of 8YF6, 8YF7, and 8YF8; Cryo-EM maps of the GNNV virion at pH 6.5 and 5.0 have EMDB accession numbers 39215 and 39217, and the atomic model of the GNNV virion at pH 6.5 is with PDB entry 8YF9. ^1^H, ^13^C, and ^15^N chemical shift assignments of GNNV-P at pH 5.0 have been deposited to the Biological Magnetic Resonance Bank (BMRB) with Accession No. 52218. The NMR structure of the GNNV P-domain has been deposited to the Protein Data Bank (PDB entry 8XID).

## Supporting Information Available

Experimental methods; Figures S1–S21; Tables S1 and S2 (PDF)
Conformational change of GNNV VLP from pH 8.0 to 5.0 (Movie S1) (MP4)
Enlarged views of GNNV protrusion conformational change from pH 8.0 to 5.0 (Movie S2) (MP4)
Hypothetical atomic model of GNNV-P conformational change from pH 8.0 to 5.0 (Movie S3) (MP4)
