# Supplementary material for: Molecular Mechanism of pH-Induced Protrusion Configuration Switching in Piscine Betanodavirus Implies a Novel Antiviral Strategy
Source: ACS Infect Dis. 2024 Aug 1;10(9):3304–19. doi: 10.1021/acsinfecdis.4c00407 (PMC11406519; doi:10.1021/acsinfecdis.4c00407)
Supplement: Supplementary file 1 — id4c00407_si_001.pdf [file id4c00407_si_001.pdf]

Supporting Information

Molecular mechanism of pH-induced protrusion configuration switching in piscine betanodavirus implies novel anti-viral strategy

Petra Štěrbová [1,2,3], Chun-Hsiung Wang [3,#], Kathleen J.D. Carillo [3,†], Yuan-Chao Lou [4], Takayuki Kato[5,§], Keiichi Namba[5], Der-Lii M. Tzou [3], and Wei-Hau Chang [1,3,6,*]

[1] Chemical Biology and Molecular Biophysics Program, Taiwan International Graduate Program, Academia Sinica, Taipei 11529, Taiwan

[2] College of Life Science, National Tsing Hua University, Hsinchu 30044, Taiwan

[3] Institute of Chemistry, Academia Sinica, Taipei 11529, Taiwan

[4] Biomedical Translation Research Center, Academia Sinica, Taipei 11529, Taiwan

[5] Graduate School of Frontier Biosciences, Osaka University, 1-3 Yamadaoka, Suita, Osaka, 565-0871, Japan

[6] Institute of Physics, Academia Sinica, Taipei 11529, Taiwan

*To whom correspondence may be addressed. Email: weihau@gate.sinica.edu.tw (Wei-Hau Chang).

Present Addresses:

[#]Academia Sinica Cryo-EM Facility, Academia Sinica, Taipei, Taiwan

[†]Institute for Bioscience and Biotechnology Research, Univeristy of Maryland, College Park, Maryland

[§]Institute of Protein Research, Osaka University, Suita, Osaka, 565-0871, Japan

This file includes:

- **Experimental Methods**
- **Figure S1 to S21**
- **Table S1, S2**
- **Caption for Movie S1, S2, and S3**
- **References**

Other supporting Information for this manuscript includes the following:

Movie S1. Conformational change of GNNV VLP from pH 8.0 to 5.0. A video showing density map and conformational change of wild type DGNNV virus-like particle from pH 8.0 to 5.0.

Movie S2. Enlarged views of GNNV protrusion conformational change from pH 8.0 to 5.0. A video showing DGNNV protrusion domain density map and DGNNV protrusion domain conformation change in pH 8.0, pH 6.5, and pH 5.0.

Movie S3. Hypothetical atomic model of GNNV-P conformational change from pH 8.0 to 5.0. A video showing conformational change of each subunit of DGNNV capsid protein in pH 8.0, pH 6.5, and pH 5.0.

Experimental Methods

Sample preparation of GNNV and VLPs for cryo-EM

GNNV and VLPs were purified using a 10-40% (w/w) sucrose density gradient, as previously described.[1,2] To prepare GNNV and VLPs in different pH conditions, the purified particles were pelleted down by ultra-centrifugation at 30,000 rpm for 3.5 hours at 4 ℃ (Beckman Coulter, OptimaTM L-90K Ultracentrifuge, rotor: SW 41 Ti). The particle pellets were then re-suspended overnight in 100 µl of TN buffer (50 mM NaCl, 50 mM Tris-HCl, pH 8.0), MES buffer (50 mM NaCl, 50 mM MES, pH 6.5), or acetate buffer (50 mM NaCl, 50 mM sodium acetate, pH 5.0), respectively.

To prepare the cryo-EM samples of GNNV and VLPs, approximately 3.5 µl of protein solution was deposited onto a Quantifoil R1.2/1.3 holey carbon grid (Quantifoil Micro Tools GmbH, Jena, Germany) coated with a thin carbon film. The grid was then rapidly plunged into liquid nitrogen-cooled liquid ethane and stored in liquid nitrogen until imaging. The cryo-EM grids of GNNV at pH 6.5 and pH 5.0 were prepared using a Vitrobot Mark IV system (Thermo Fisher Scientific, Hillsboro, OR, USA) at 4 ℃ and 100% humidity, with a blotting time of 3.5 seconds. For VLPs at pH 8.0, pH 6.5 and pH 5.0, the cryo-EM grids were prepared using a Leica EM GP system (Leica Biosystems, Deer Park, IL, USA) with the sensor off option—the cryo-EM grids of VLPs at pH 8.0 and pH 5.0 were prepared at 15 ℃ and 80% humidity, with a blotting time of 1.2 seconds; whereas the cryo-EM grids of VLPs at pH 6.5 was prepared at 22 ℃ and 95% humidity, with a blotting time of 0.5 seconds. All subsequent steps were conducted at liquid nitrogen temperature to prevent de-vitrification.

Cryo-EM data acquisition

The cryo-EM grids containing GNNV virions at pH 6.5 and pH 5.0 were examined using cryo-ARM (JEOL Ltd., Akishima, Tokyo, Japan) at magnifications of 40,000x and 50,000x, respectively, with pixel sizes of 1.36 Å/pixel and 1.09 Å/pixel. Cryo-EM images of GNNV at pH 6.5 and pH 5.0 were recorded using a K2 camera (Gatan Inc., Pleasanton, CA, USA) in counting mode, with an exposure time of 8 seconds for 40 frames. The dose rate was approximately 6.5 electrons/Å^2 per second, resulting in a total accumulated dose of around 52 electrons/Å^2 (equivalent to approximately 1.3 electrons/Å^2 per frame).

The cryo-EM grids containing VLPs at pH 6.5 were examined using Technai F20 (FEI, Hillsboro, OR, USA) at a nominal magnification of 29,000x, resulting in a pixel size of 1.24 Å/pixel. Cryo-EM images of the VLPs at pH 6.5 were recorded using a K2 camera (Gatan Inc., Pleasanton, CA, USA) in counting mode, with an exposure time of 10 seconds for 50 frames. The dose rate was approximately 5 electrons/Å^2 per second, resulting in a total accumulated dose of around 50 electrons/Å^2 (equivalent to approximately 1.0 electron/Å^2 per

frame).

The cryo-EM grids containing VLPs at pH 8.0 and pH 5.0 were examined using JEM-2100F with a high-contrast pole piece (JEOL Ltd., Akishima, Tokyo, Japan) at a magnification of 50,000x, with a pixel size of 1.16 Å/pixel. Cryo-EM images of the VLPs at pH 8.0 and pH 5.0 were recorded using a DE-20 camera (Direct Electron LP, San Diego, CA, USA) in linear mode, with an exposure time of 1.5 seconds for 38 frames. The dose rate was approximately 20 electrons/$Å^2$ per second, resulting in a total accumulated dose of around 30 electrons/$Å^2$ (equivalent to approximately 0.8 electrons/$Å^2$ per frame). The parameters for cryo-EM data acquisition are summarized in **Table S1**.

Single-particle image processing and 3D reconstruction

All cryo-EM image stacks underwent motion correction and dose weighting using MotionCor2[3] with a 5 x 5 patch. The contrast transfer function (CTF) was determined using CTFFIND4[4] from the motion-corrected and dose-weighted images. Particle picking was conducted in cryoSPARC[5] using 2D templates generated from a previously determined VLP cryo-EM map.[2] After particle extraction and removal of bad particles through 2D classification, the remaining particles were used for further *ab initio* reconstruction and heterogeneous refinement with icosahedral symmetry (I). A subset of particles from a good 3D class with more particles and better resolution were chosen for homogeneous refinement with icosahedral symmetry (I). Overall resolution was assessed using the Fourier Shell Correlation (FSC) = 0.143 criterion, and local resolution was calculated within cryoSPARC[5]. The final cryo-EM maps achieved overall resolutions of 3.12 Å (GNNV pH 6.5), 4.36 Å (GNNV pH 5.0), 3.23 Å (VLP pH 8.0), 2.82 Å (GNNV pH 6.5), and 3.52 Å (GNNV pH 5.0), respectively (**Figure S3, S4, and Table S1**). Visualization of the resulting 3D density maps was performed using UCSF Chimera.[6] The details of single-particle image reconstructions of GNNV and VLPs can be found in the flowcharts in **Figure S21 and S22**, and the cryo-EM reconstruction details are summarized in **Figure S3 and S4**. Additional information regarding cryo-EM reconstruction statistics is available in **Table S1**.

Protein construct and site-directed mutagenesis

The cDNA encoding the Dragon grouper NNV P-domain (aa 214-338, GNNV-P) was tagged with His_6-yeast SUMO (Smt3) at the N-terminus and was cloned into pETDuet-1 vector. GNNV-P mutant constructs—namely R276A, W280A, H281Y, W301A, Q322A, I323A, L324A, and L325A—were created using plasmids harboring the wild type GNNV-P sequence and a QuickChange Lightning site-directed mutagenesis kit (Agilent Technologies, CA, USA). Mutations were confirmed by PCR sequencing (Genomics Inc., Taiwan). Primers used for mutagenesis were synthesized by Tri-I Biotech Inc. (NTC, Taiwan).

Protein expression and purification

Wild type and mutant GNNV-P proteins were expressed using transformed *Escherichia coli* BL21 (DE3) strain according to expression and purification protocols reported previously.[7] U-[^{2}H, ^{13}C, ^{15}N] triple-labeled proteins for NMR assignments at pH 5.0 were expressed in M9 minimal medium prepared using 100% D_2O (Sigma-Aldrich) as solvent (M9 D_2O medium), with 1 g/L $^{15}NH_4Cl$ (Sigma-Aldrich) and 2 g/L U-$^{13}C_6$-Glucose (Cambridge Isotope Laboratories) as the sole nitrogen and carbon sources, respectively, according to a modified expression protocol. In brief, overnight culture of transformed *E. coli* was first transferred to 1L of LB medium supplemented with 100 μg/ml ampicillin and grown until the OD_{600} reached a value of 1.0. The cells were then collected by centrifugation and excess LB medium was removed before re-suspending the cells in M9 D_2O medium. Protein expression was induced by addition of IPTG (final concentration of 0.3 mM) dissolved in 100% D_2O. Then, the cells were grown for an additional 4 hours at 37 °C with shaking at 150 rpm, before being harvested by centrifugation at 8000 rpm for 25 minutes at 4 °C.

Sedimentation velocity analytical ultracentrifugation (SV AUC)

The sedimentation velocity experiments were performed in a Beckman Coulter ProteomeLab XL-I analytical ultracentrifuge equipped with a 190-800 nm absorbance optical system. The concentration of protein samples used for SV AUC was in the 0.25 - 0.4 mg/ml range. The SV experiments were carried out at a rotor speed of 60,000 rpm at 20 °C and absorbance was monitored at λ = 280 nm. Protein sample buffer was used as a reference. Sedimentation coefficients were determined using the SEDFIT v16-1c software.[8] The sedimentation velocity data was fitted into a continuous c(s) distribution model based on solving the Lamm equation by the least-squares technique.[8] Buffer density (ρ), viscosity (η), and molecule partial specific volume were estimated using SEDNTERP software.[9]

NMR spectroscopy and structure determination

All NMR experiments were performed at 298 K on Bruker AVANCE 600, 800, and 850 MHz spectrometers equipped with 5 mm triple resonance TXI cryogenic probes including a shielded Z-gradient. Samples containing 10% D_2O were loaded into 5 mm Shigemi NMR tubes for NMR experiments.

The sequence-specific backbone resonance assignments at pH 5.0 were achieved using 1.0 mM of U-[^{2}H, ^{13}C, ^{15}N]-labeled GNNV-P protein in 20 mM sodium acetate (pH 5.0), 50 mM NaCl, 0.5 mM ethylenediaminetetraacetic acid (EDTA), 0.02% sodium azide, and 90% H_2O/10% D_2O. NMR spectra were processed using Bruker Topspin 3.6 and analyzed using NMRviewJ 9.2.0.[10] The sequence-specific backbone assignments have been determined by independent connectivity analysis of HNCACB, HNCO and HN(CA)CO experiments. We

completed backbone assignments for 123 out of 125 residues (98.3%), with the exceptions of Thr214 and Leu325. These resonance assignments have been deposited into the Biological Magnetic Resonance Databank with accession code 52218.

GNNV-P structural calculations at neutral pH were carried out in XPLOR-NIH software version 3.8[11] in NMRbox[12] using experimentally determined distance restraints, hydrogen bonds, and predicted dihedral angles. Backbone and side-chain NMR assignments of the NNV P-domain at neutral pH were reported previously.[7] NOE distance restraints were derived from an ^{15}N-edited NOESY-HSQC spectrum and they were analyzed using NMRviewJ software.[10] Hydrogen bonds were identified based on hydrogen/deuterium exchange experiments. The Backbone dihedral angle restraints Φ and Ψ were predicted from chemical shifts using the TALOS+ webserver.[13] For GNNV-P structural determination, 100 structures were generated according to a standard simulated annealing protocol. Twenty structures with the lowest energy were selected for refinement using the implicit solvation potential and effective energy function (EFFx) in XPLOR-NIH.[14] The twenty structures with the lowest energy and without reported violations were selected for assessment and quality checking using the protein structure validation suite in the wwPDB Validation server.[15] The final ensemble of 20 structural conformations of GNNV-P has been deposited in the Protein Data Bank (PDB entry 8XID) and the respective structural statistics are summarized in **Table S2**.

Amide hydrogen-deuterium exchange rate (HXD)

Hydrogen-deuterium exchange between amide NH signal and D_2O solvent was monitored using ^{1}H, ^{15}N-HSQC spectra. First, an initial ^{15}N-HSQC spectrum was collected before freezing GNNV-P in liquid nitrogen and lyophilizing it. The progress of amide hydrogen-deuterium exchange was monitored by collecting ^{15}N-HSQC spectra for GNNV-P samples at pH 7.0 after re-dissolving them in 100% D_2O for 4 hours. The hydrogen-deuterium exchange rate was calculated by fitting peak intensities into the exponential decay function I(t)=I_0·e^(-t/x) using NMRviewJ.[10]

Chemical shift perturbation and secondary chemical shifts calculation

2D ^{1}H-^{15}N HSQC spectra of GNNV-P at pH 7.0 and 5.0 were used for chemical shift perturbation analysis. The chemical shift between pH 7.0 and pH 5.0 was calculated using the equation $\Delta\delta = \sqrt{\Delta\delta_H^2 + (\alpha \cdot \Delta\delta_N)^2}$, where $\Delta\delta_H$ and $\Delta\delta_N$ are the ^{1}H and ^{15}N chemical shift changes, respectively. A scaling factor of α = 0.1 was used to account for the larger ^{15}N chemical shift.[16] Secondary structure propensities were predicted from $^{13}C_\alpha$ and $^{13}C_\beta$ chemical shifts, based on their deviations from random coil values.[17]

Molecular dynamics (MD) simulation

Simulations for GNNV-P trimer formation were performed using the GROMACS package.[18]

Initially, protein protonation state was adjusted to pH 5.0 using PDB2PQR software.[19] To prepare the initial point for the simulation, three protein molecules were initially packed in a ~300 Å cubic box using the software PACKMOL.[20] This step was done to ensure that no repulsive interactions would disrupt or cause an error during the simulations. Using a V-rescale thermostat, the overall temperature of the water and protein were kept constant by coupling each group of molecules independently at 300 K. A Parrinello-Rahman barostat was used to separately couple the pressure to 1 atm in every dimension.[21] The time constants for the temperature and pressure coupling were set to 0.1 and 2 ps, respectively. A time step of 2 fs was applied using the leapfrog algorithm to integrate the equations of motion for the system. Periodic boundary conditions were set for the whole system. For the Lennard–Jones and the Ewald sum Coulombic interactions, we set a 1 nm cut-off. The Fourier space part of the Ewald splitting was calculated using the particle-mesh-Ewald method, by applying cubic spline interpolation and 0.16 nm grid length on the side.[22] The TIP3P water model was used and the protein parameters were obtained from the AMBERff99SB-ILDN force field.[23,24] MD simulations were done for a total scan length of 100 ns.

Molecular docking analysis

Molecular docking analysis on GNNV-P with two sialoside isomers, Neu5Ac-(α2,3)-Lac and Neu5Ac-(α2,6)-Lac, was carried out using the HADDOCK 2.4 webserver and protein-glycan default settings.[25] The three-dimensional structures of Neu5Ac-(α2,3)-Lac and Neu5Ac-(α2,6)-Lac were extracted from PDB entries 6TLZ and 6TM0, respectively. The binding energy between GNNV-P and the docked sialosides was estimated using the PRODIGY webserver.[26]

SV-AUC data interpretation and protrusion hydrophobicity analysis

Interpretations of pH 6.0 and 5.5 SV-AUC profile were performed with cautions according to Schuck and Zhao.[27] Hydrophobicity patches on P-domain (pH 7.0) and protrusion as trimer of P-domains (pH 5.0) were analyzed using Kyte-Dolittle analysis.[28]

Figure S1. Cryo-EM images of GNNV in different pH environments.

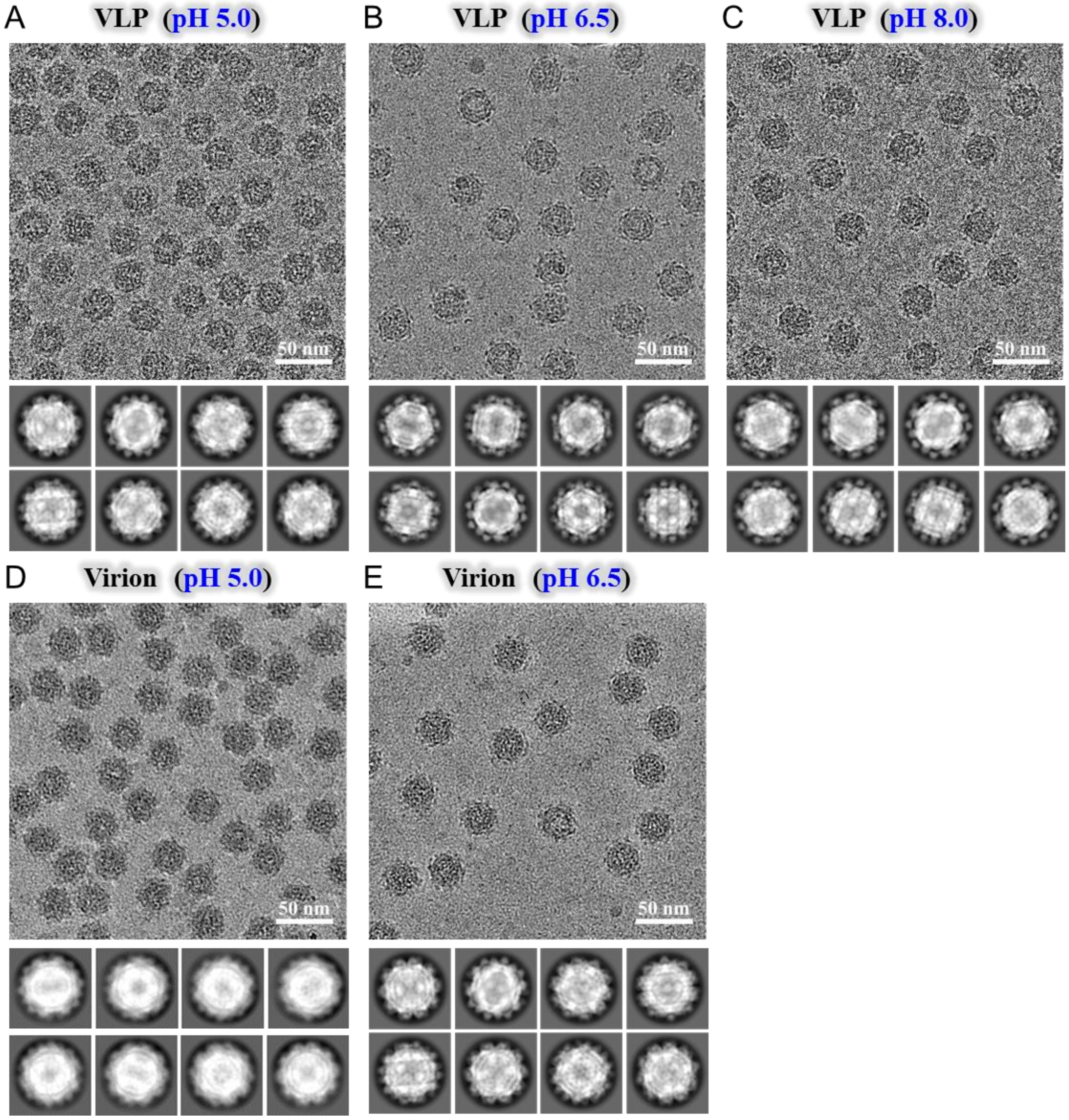

Supplementary Figure 1. Cryo-EM micrographs class averages of GNNV in different pH environments. (A) GNNV VLP at pH 5.0. bar: 50 nm. **(B)** GNNV VLP at pH 6.5. bar: 50 nm. **(C)** GNNV VLP at pH 8.0. bar: 50 nm. **(D)** GNNV virion at pH 5.0. bar: 50 nm. **(E)** GNNV virion at pH 6.5. bar: 50 nm.

Figure S2. Structure of native GNNV virions compared to virus-like particles (VLP) at the same pH.

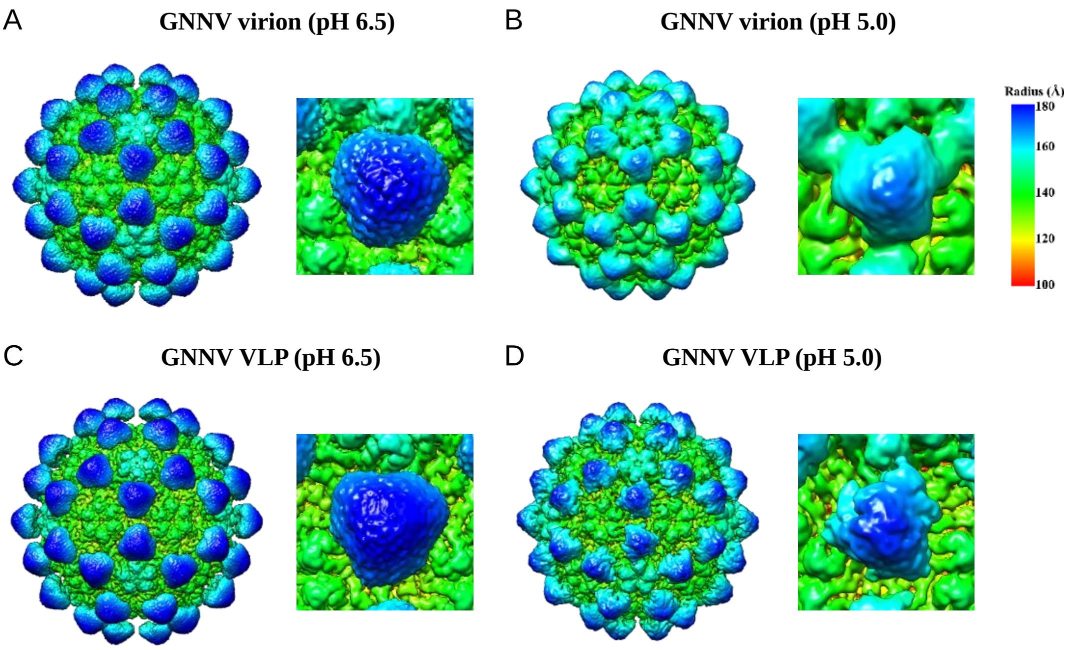

Supplementary Figure 2. Structure of native GNNV virions compared to virus-like particles (VLP) at the same pH. (A) GNNV virion at pH 6.5 (resolution 3.12Å , contours at 2σ). **(B)** GNNV virion at pH 5.0 (resolution 4.36Å, contours at 3σ). **(C)** GNNV VLP at pH 6.5 (resolution 2.72Å , contours 2σ). **(D)** GNNV VLP at pH 5.0 (resolution 3.65Å , contours at 2σ). The structure of GNNV is colored from red to blue according to the radius, as shown by the color bar. In the panels at right of A-D, a magnified view of a protrusion is shown. Note that the VLP data at pH 6.5 was collected using an F20 electron microscope (FEI, Hillsboro, OR, USA) with a K2 camera (Gatan Inc., Pleasanton, CA, USA), whereas the VLP data at pH 5.0 was collected using a JEM-2100F electron microscope (JEOL Ltd., Akishima, Tokyo, Japan) with a DE-20 camera (Direct Electron LP, San Diego, CA, USA).

Figure S3. Cryo-EM structural determination of GNNV VLPs at H8.0, pH 6.5 and pH 5.0.

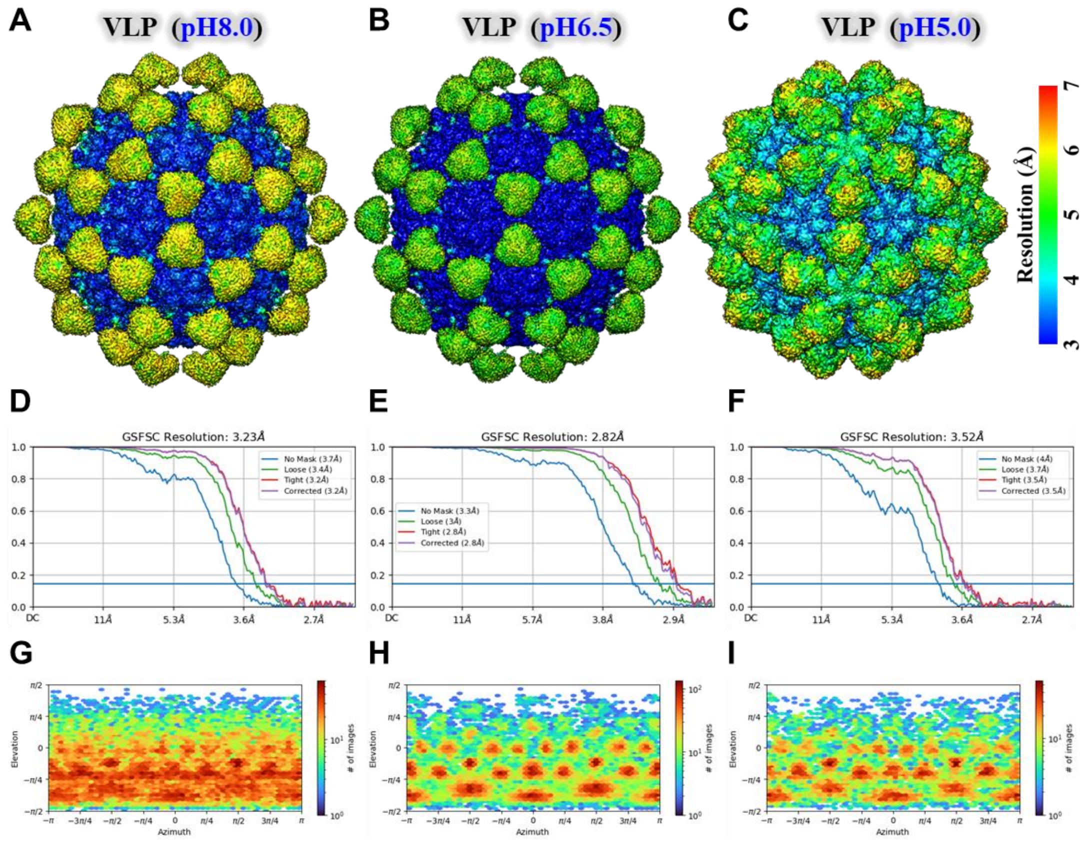

Supplementary Figure 3. Cryo-EM structural determination of GNNV VLPs at H8.0, pH 6.5 and pH 5.0. Local resolution analysis of the cryo-EM maps of GNNV VLPs at **(A)** pH 8.0, **(B)** pH 6.5, and **(C)** pH 5.0. The cryo-EM maps of GNNV VLPs are colored according to the resolution, as shown by the color bar. Gold-standard FSC curves (FSC= 0.143) of the cryo-EM maps of GNNV VLPs at **(D)** pH 8.0, **(E)** pH 6.5, and **(F)** pH 5.0. The angular distributions of all particle projections in the final 3D reconstructions of GNNV VLPs at **(G)** pH 8.0, **(H)** pH 6.5, and **(I)** pH 5.0. The heat maps depict the number of particles observed for each viewing angle. Regions colored in red indicate a higher particle count, indicating a more frequent occurrence of those specific viewing angles.

Figure S4. Cryo-EM structural determination of GNNV virions at pH 6.5 and pH 5.0.

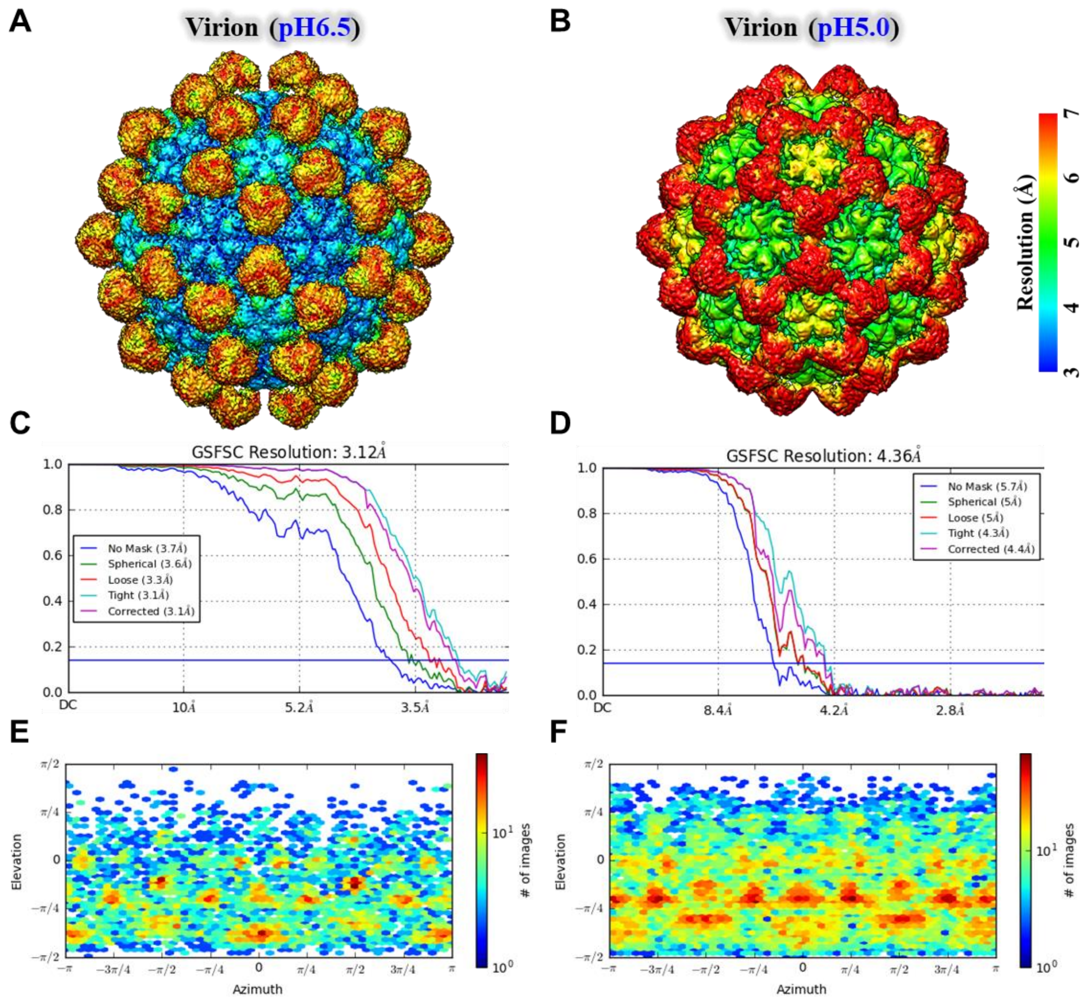

Supplementary Figure 4. Cryo-EM structural determination of GNNV virions at pH 6.5 and pH 5.0. Local resolution analysis of the cryo-EM maps of GNNV virions at **(A)** pH 6.5 and **(B)** pH 5.0. The cryo-EM maps of GNNV virion are colored according to the resolution, as shown by the color bar. Gold-standard FSC curves (FSC= 0.143) of the cryo-EM maps of GNNV virions at **(C)** pH 6.5 and **(D)** pH 5.0. The angular distributions of all particle projections in the final 3D reconstructions of GNNV virions at **(E)** pH 6.5 and **(F)** pH 5.0. The heat maps depict the number of particles observed for each viewing angle. Regions colored in red indicate a higher particle count, indicating a more frequent occurrence of those specific viewing angles.

Figure S5. Particle diameters of GNNV VLPs at different pH and the radial density distribution of GNNV VLPs.

A

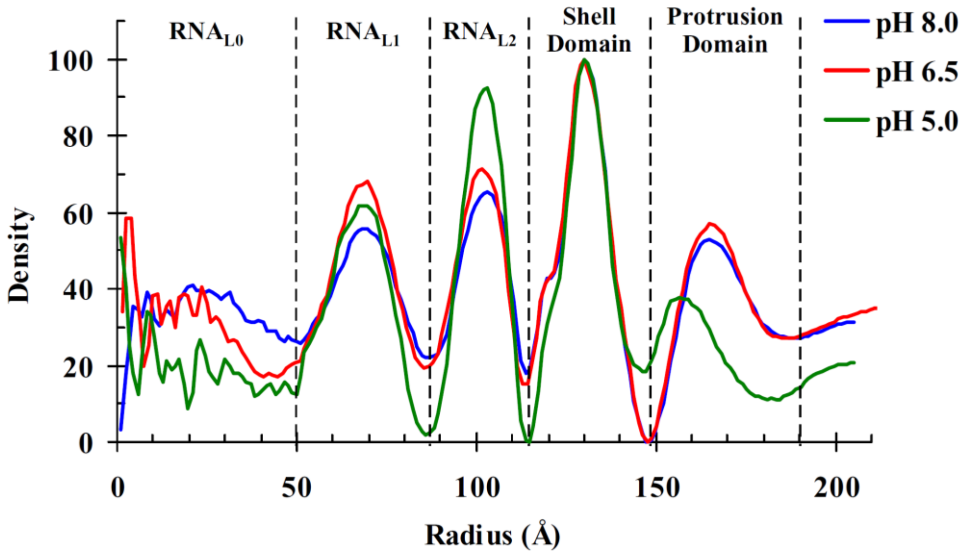

Supplementary Figure 5. Particle diameters of GNNV at different pH and radial density distribution of GNNV. (A) The surface view of the GNNV particles at pH8.0 (*left panel*), pH6.5 (*middle panel*), and pH5.0 (*right panel*). The maps are colored based on the radius. **(B)** The radial density distribution of GNNV VLPs in various pH environments. Three sections correspond to the protrusions (~149 to 190 Å), the capsid shell (~115 to 149 Å).

Figure S6. Cross-sections of GNNV VLPs in three different pH environments.

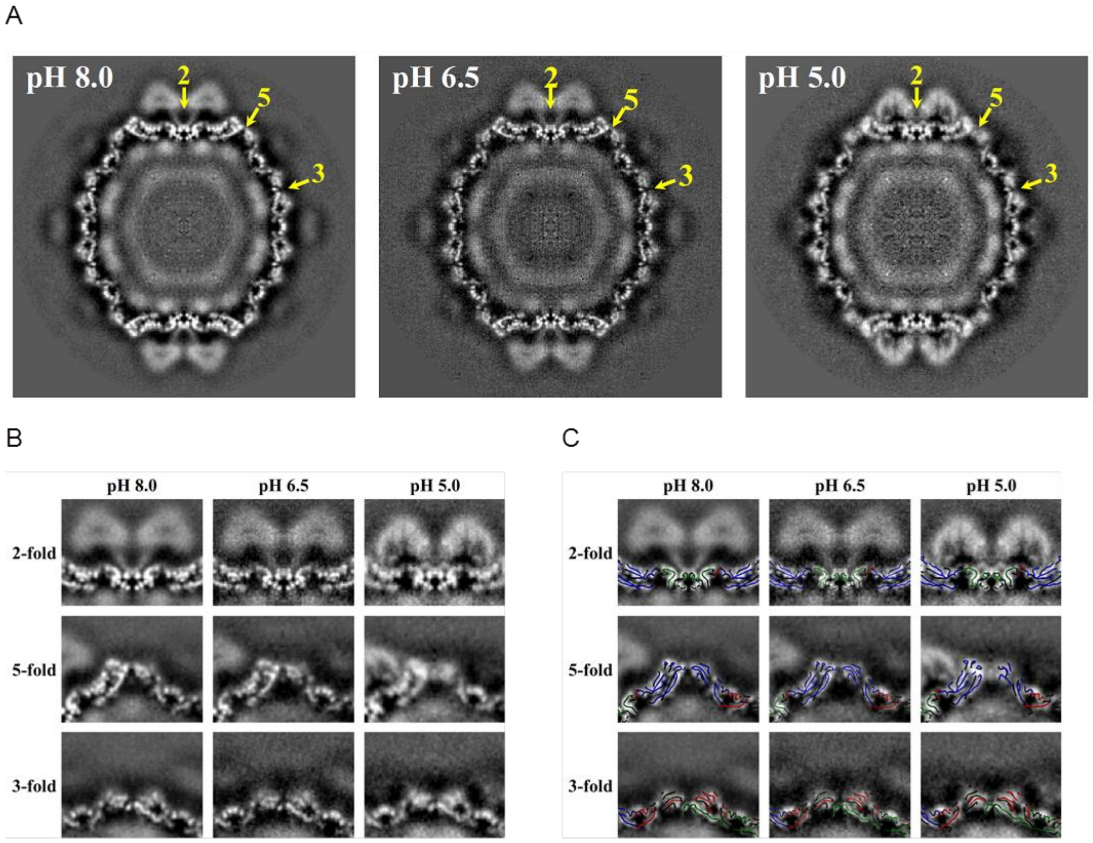

Supplementary Figure 6. Cross-sections of DGNNV VLPs in three different pH environments. (A) Cross sections of a GNNV particle at pH 8.0 (*left panel*), pH 6.5 (*middle panel*), and pH 5.0 (*right panel*). Icosahedral 2-, 5- and 3-fold axes are indicated. **(B)** Enlarged views of the cross sections around icosahedral 2-, 5- and 3-fold axes in (A). **(C)** Fitting of atomic models of the S-domain subunits A, B, and C (colored blue, red and green, respectively) into the cross sections in (B).

Figure S7. NMR spectra of deuterium-labeled GNNV-P at pH 5.0.

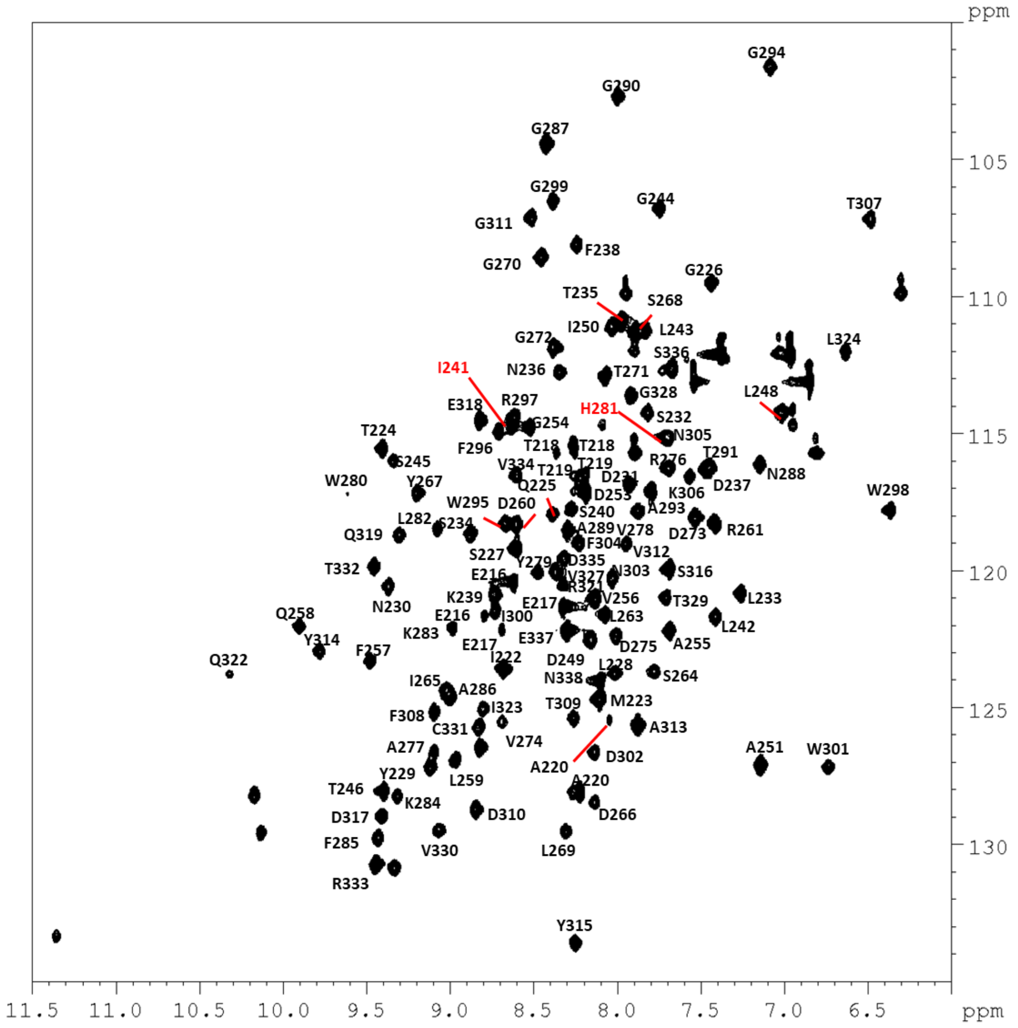

Supplementary Figure S7. NMR spectra of deuterium-labeled GNNV-P at pH 5.0. HSQC spectra of ^{2}H-, ^{13}C-,and ^{15}N-labeled GNNV-P recorded at pH 5.0. The resonance assignments for pH 5.0 have been deposited into the Biological Magnetic Resonance Databank with Accession No. 52218.

Figure S8. NMR spectra reveal the effects of pH on GNNV-P.

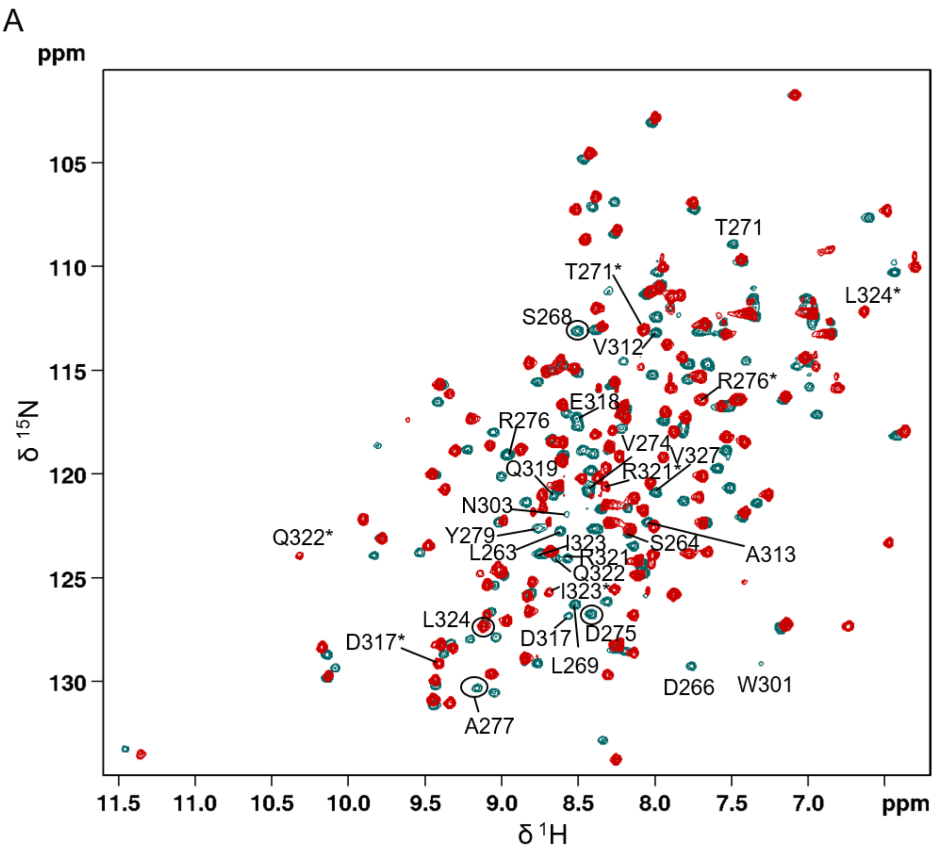

B

```
TPEETTAPIMTQGSLYNDSLSTNDFKSILLGSTPLDIAPD  40
GAVFQLDRPLSIDYSLGTGDVDRAVYWHLKKFAGNAGTPA  80
GWFRWGIWDNFNKTFTDGVAYYSDEQPRQILLPVGTVCTR 120
VDSEN                                    125
```

Supplementary Figure S8. (Superposition and highlight missing ones and 4 with highest CSP) NMR spectra reveal the effects of pH on GNNV-P. **(A)** Superimposition of ^{1}H-^{15}N HSQC spectra of ^{15}N-labeled GNNV-P recorded at pH 7.0 (blue) and ^{2}H, ^{13}C, ^{15}N-labeled GNNV-P recorded at pH 5.0 (red). Resonances of the pH-sensitive peaks at pH 7 are labeled. New positions for residues with the greatest chemical shift perturbations (CSP) at pH 5.0 are marked by *. **(B)** GNNV-P amino acid sequence with pH-sensitive residues highlighted in blue. Red residues represent those displaying the greatest CSPs between neutral and acidic pH.

Figure S9. Sedimentation velocity analytical ultracentrifugation analysis of GNNV-P oligomerization.

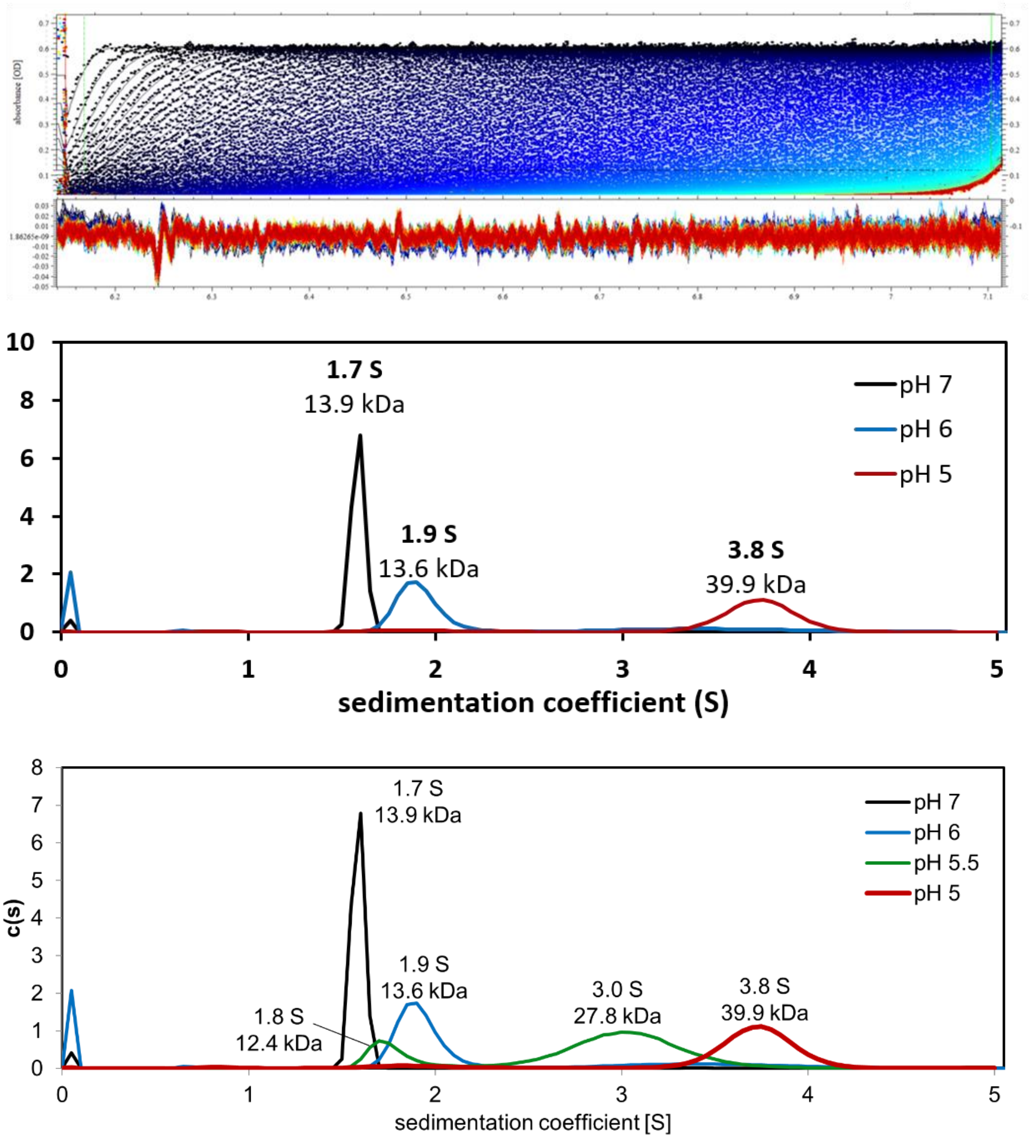

Supplementary Figure S9. Sedimentation velocity analytical ultracentrifugation analysis of GNNV-P oligomerization. Plot of the sedimentation velocity analytical ultracentrifugation (SV AUC) data fitted to a continuous sedimentation coefficient distribution c(s) model. The GNNV-P sedimentation coefficients were determined at pH 7.0 (black), 6.0 (blue), and 5.0 (red). Sedimentation coefficients and estimated molecular weights are noted above each peak. Representative raw sedimentation profile of absorbance at 280nm and representative residuals from fitting the data to a continuous c(s) distribution model are shown in the panels above the plot. Note that the estimated molecular weights of 13.6 kDa for 1.9 S in the pH 6 profile and 12.4 kDa for 1.8 S in the pH 5.5 profile are considered to be artifacts due to the modeling based on the assumption of single species. [27]

Figure S10. Hydrogen-deuterium exchange rate (HXD) of GNNV-P at pH 7.0.

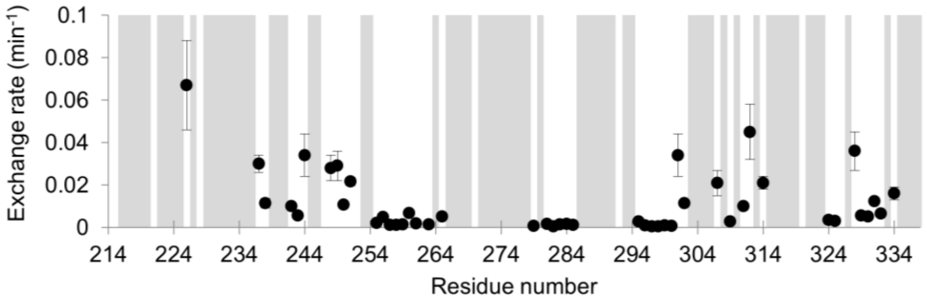

Supplementary Figure S10. Hydrogen-deuterium exchange rate (HXD) of GNNV-P at pH 7.0. Plot of the hydrogen-deuterium exchange rate determined at neutral pH as a function of protein sequence. Residues for which HXD could not be measured (i.e., exchanging faster than our detection limits) are indicated by a grey background.

Figure S11. Mapping of pH sensitive residues mapped on the GNNV-P trimer crystal structure.

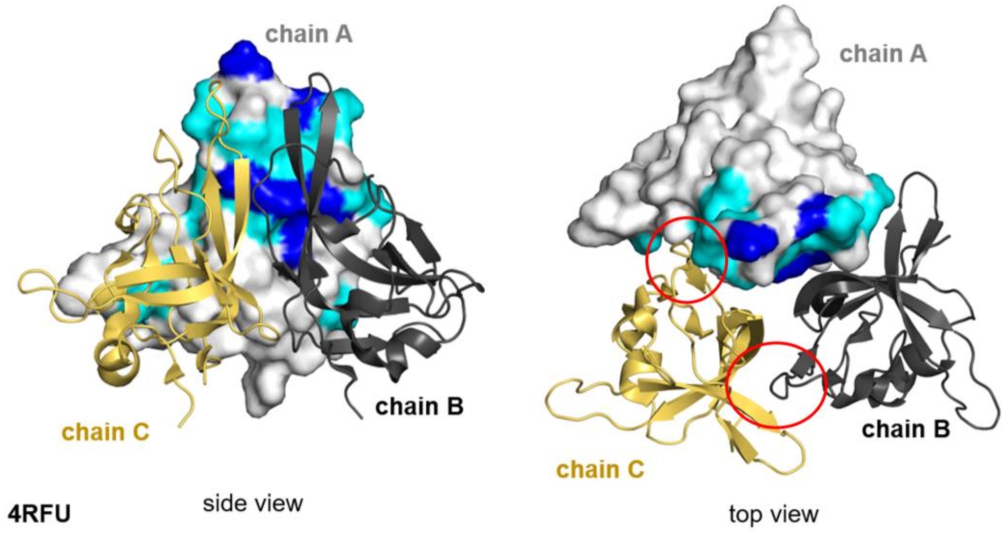

Supplementary Figure S11. Mapping of pH sensitive residues mapped on the GNNV-P trimer crystal structure. pH sensitive residues identified by NMR pH titration mapped onto the crystal structure of trimeric GNNV-P (PDB ID 4RFU). The pH sensitive residues have been mapped onto the surface representation of chain A consistent with the color scheme in Fig. 2C. Chain B and chain C are displayed as yellow and black cartoons, respectively. The F'-G' loop and β-strand region (aa 323-326) at the trimeric interface is highlighted by red circles.

Figure S12. RMSD analysis of GNNV-P trimer stability after MD simulations.

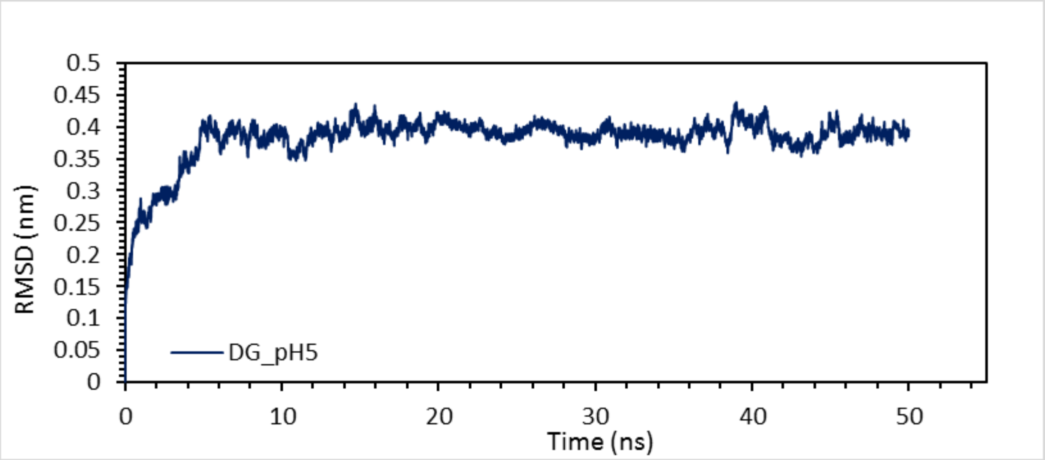

Supplementary Figure S12. RMSD analysis of GNNV-P trimer stability after MD simulations. RMSD of GNNV-P trimer during the time-course of MD simulations.

Figure S13. Comparison of the GNNV-P trimers obtained by MD simulations and X–ray crystallography.

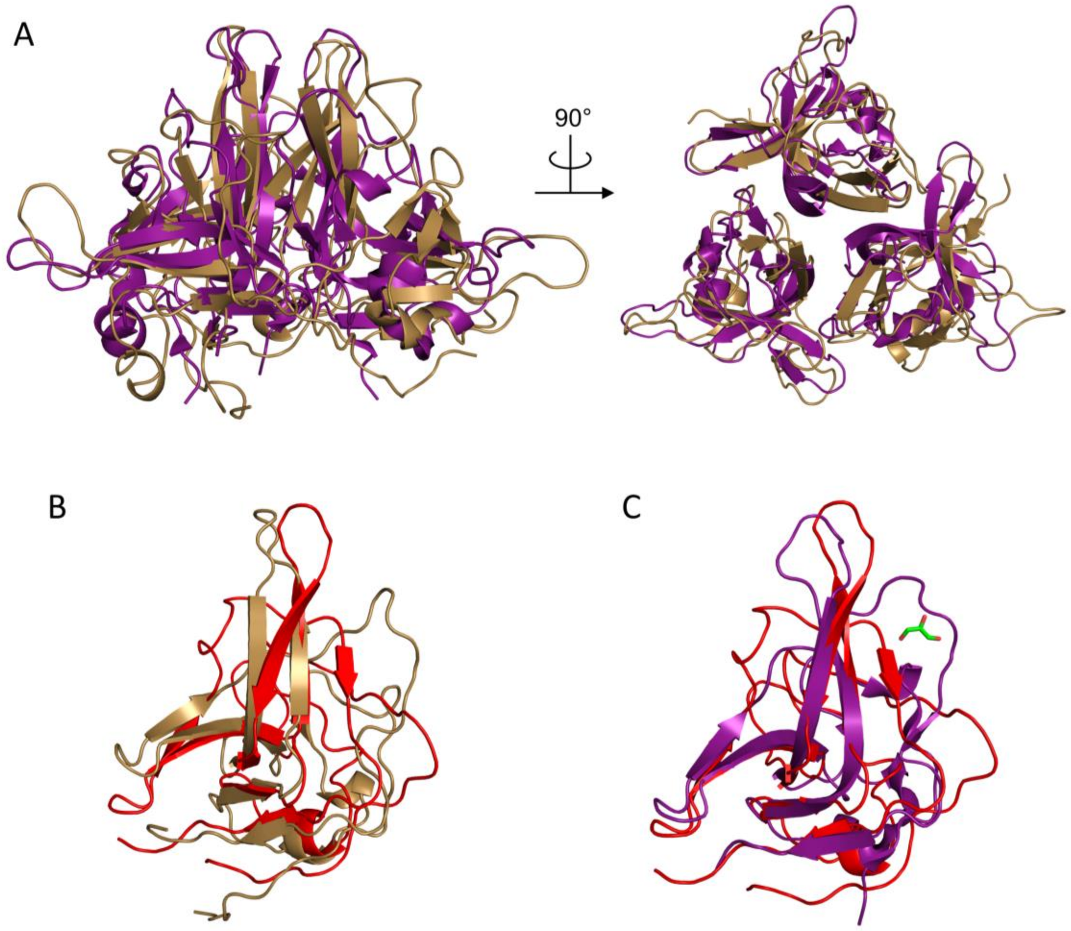

Supplementary Figure 13. Comparison of the GNNV-P trimers obtained by MD simulation and X–ray crystallography. (A) Side and top views of overlaid cartoon representations of GNNV-P trimers determined by MD simulations at pH 5.0 (purple) and X-ray crystallography at pH 6.5 (brown). (B) Superimposition of the GNNV-P structures determined in solution at neutral pH (light brown) and the model of GNNV-P at pH 5.0 obtained by MD simulations (red). (C) Superimposition of the MD-simulated GNNV-P model at pH 5.0 (red) with chain A of the GNNV-P structure determined by X-ray crystallography at pH 6.5 (purple). A conserved glycerol molecule resolved as stabilizing the F'-G' loop is shown in green as a stick model.

Figure S14. Sedimentation velocity AUC analysis of low pH-induced oligomerization of GNNV-P single mutants

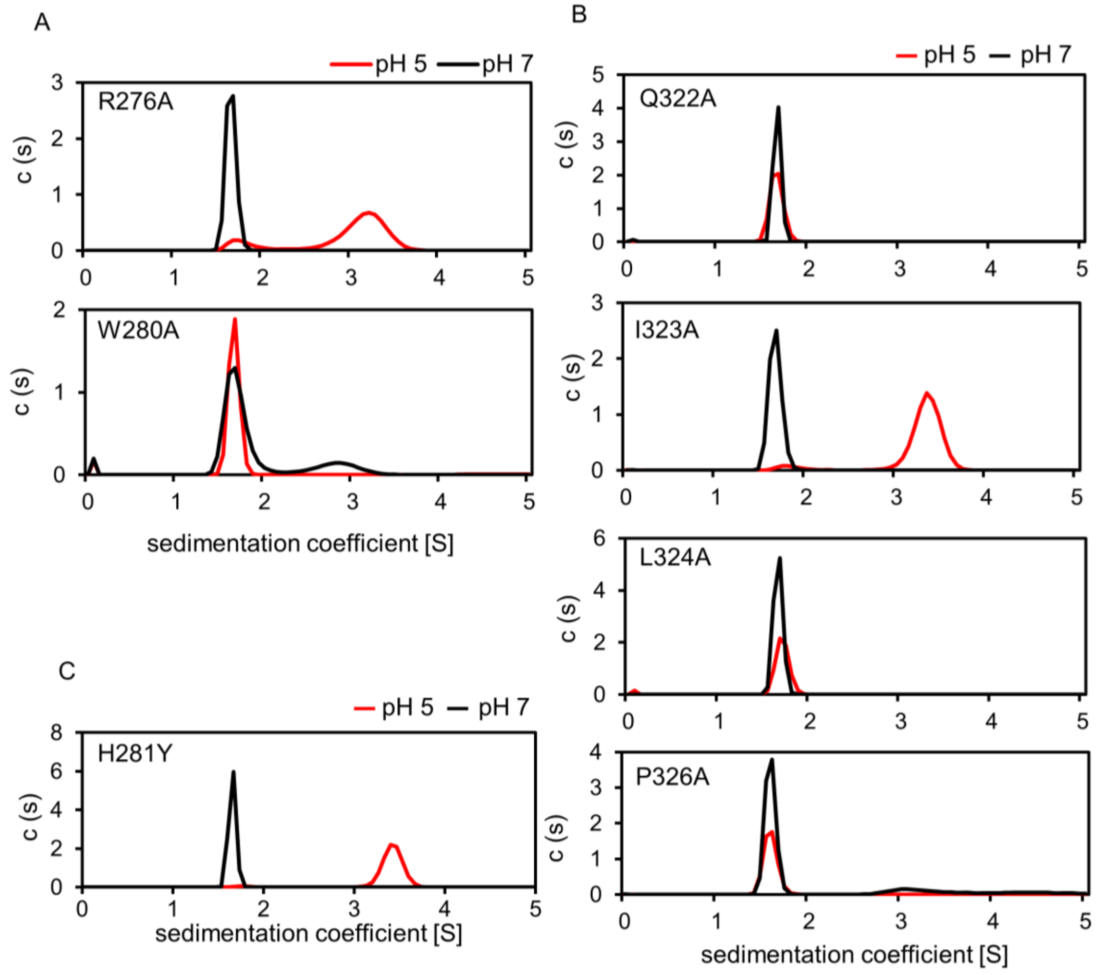

Supplementary Figure S14. Sedimentation velocity AUC analysis of low pH-induced oligomerization of GNNV-P single mutants. Plots of the sedimentation velocity analytical ultracentrifugation (SV AUC) data fitted to a continuous sedimentation coefficient distribution c(s) model for GNNV-P mutations of residues in Region 1 (A), Region III (B), and H281 (C).

Figure S15. Comparative protein sequence analysis of NNV P-domains.

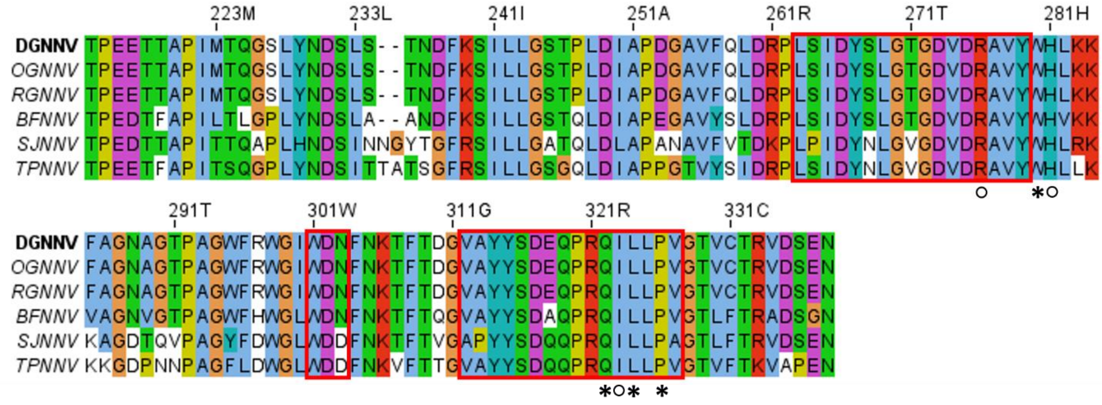

Supplementary Figure 15. Comparative protein sequence analysis of NNV P-domains. Protein sequence alignment of the P-domains of Dragon grouper nervous necrosis virus (DGNNV), Orange-spotted grouper nervous necrosis virus (OGNNV), plus four genotypic variants (RGNNV, BFNNV, SJNNV, and TPNNV), shows a high degree of conservation of amino acid residues within the F'-G' loop. pH-sensitive region I (L263-Y279), region II (W301-N303), and region III (V312-V327) are highlighted by red boxes. Residues identified by mutagenesis as being critical for GNNV-P low pH-induced oligomerization are marked by *. Residues for which mutation did not affect low pH-induced oligomerization are marked by ○.

Figure S16. Changes in the electrostatic surface potential of monomeric GNNV-P at neutral and low pH.

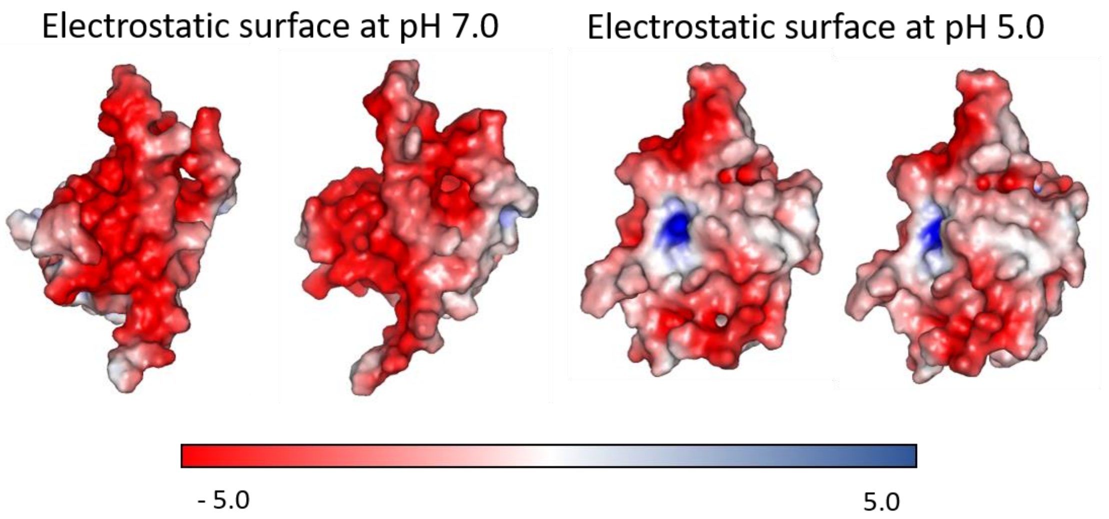

Supplementary Figure 16. Changes in the electrostatic surface potential of monomeric GNNV-P at neutral and low pH. Gradient visualization of the electrostatic surfaces from red (-5.0 kT/e) to blue (5.0 kT/e) of GNNV-P at pH 7.0 and 5.0, calculated using the APBS server based on residual pKa determined by PROPKA. The view shows the potential interface area between the A/C (left) and A/B (right) units in the GNNV-P trimer at pH 7.0 and pH 5.0.

Figure S17. The conformational change of the GNNV-P malleable linker between neutral and low pH

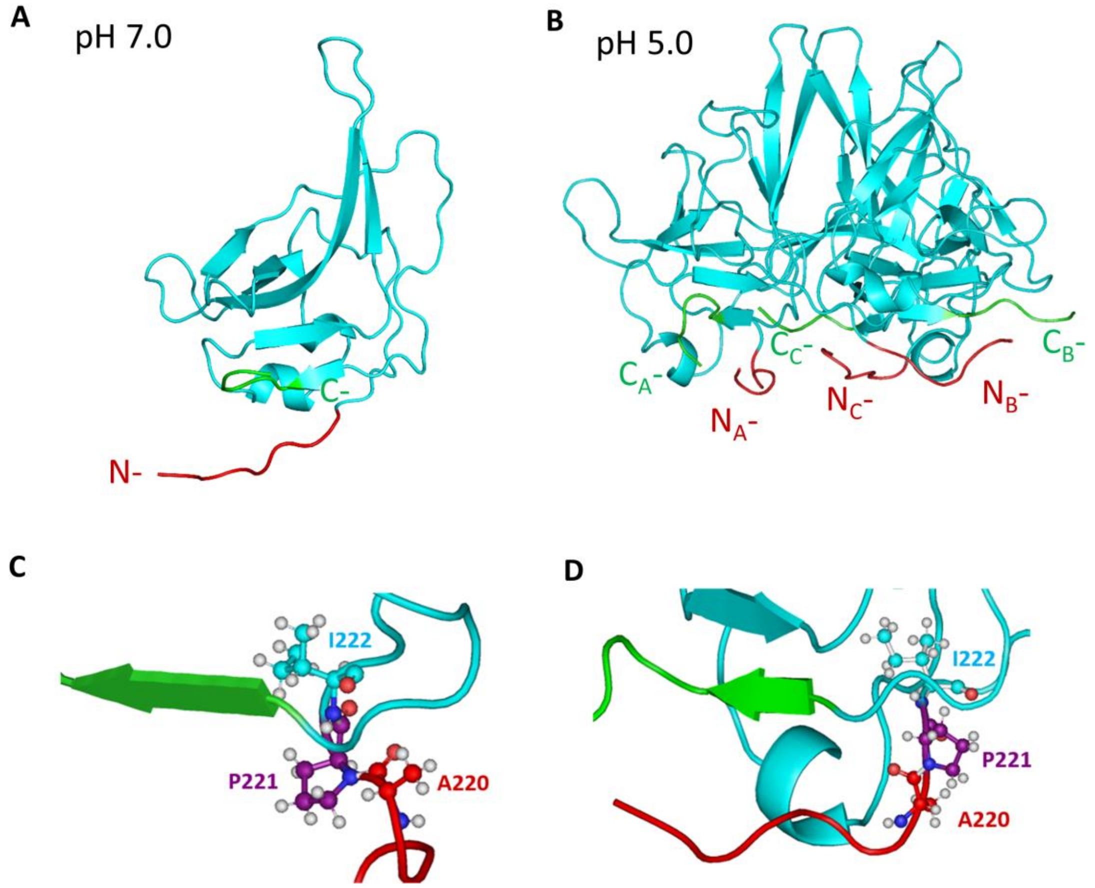

Supplementary Figure 17. The conformational change of the GNNV-P malleable linker between neutral and low pH. GNNV-P structure at neutral pH (A) and model of the GNNV-P trimer at acidic pH (B) with N- and C-terminal residues colored in red and green, respectively. Detailed view of the conformation of the malleable linker around residue P221 at neutral pH (C) and acidic pH (D). Residues A220-I222 are shown in ball and stick representation.

Figure S18. Multiple NMR assignments of residue A220 at low pH.

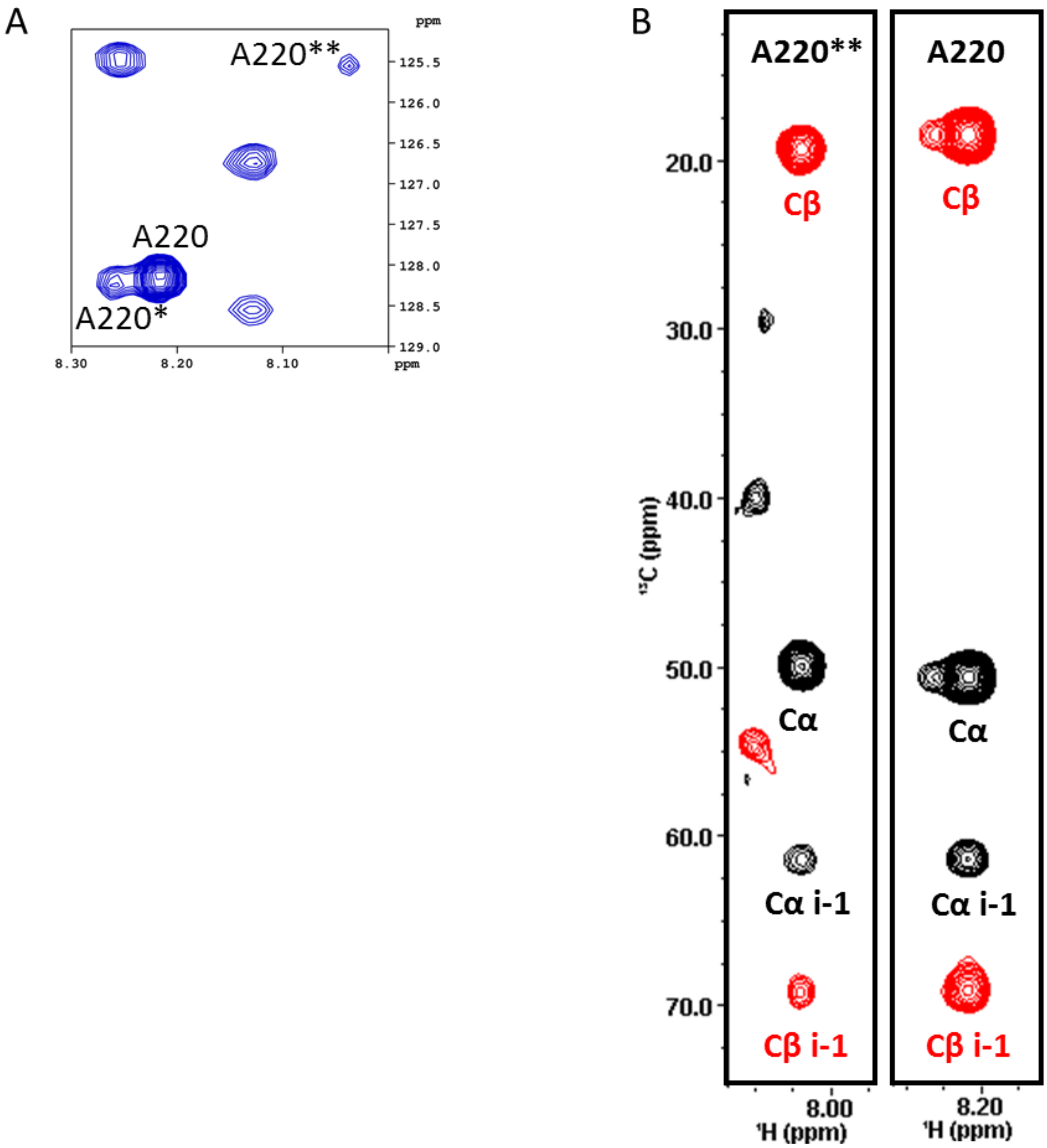

Supplementary Figure 18. Multiple NMR assignments of residue A220 at low pH.
(A) Zoomed in view of the 2D 1H-15N HSQC spectrum of GNNV-P at pH 5.0, showing multiple NMR signals for residue A220. **(B)** HNCACB spectra showing the chemical shift of residue A220.

Figure S19. Model of Neu5Ac-Lac binding to GNNV-P at pH 5.0

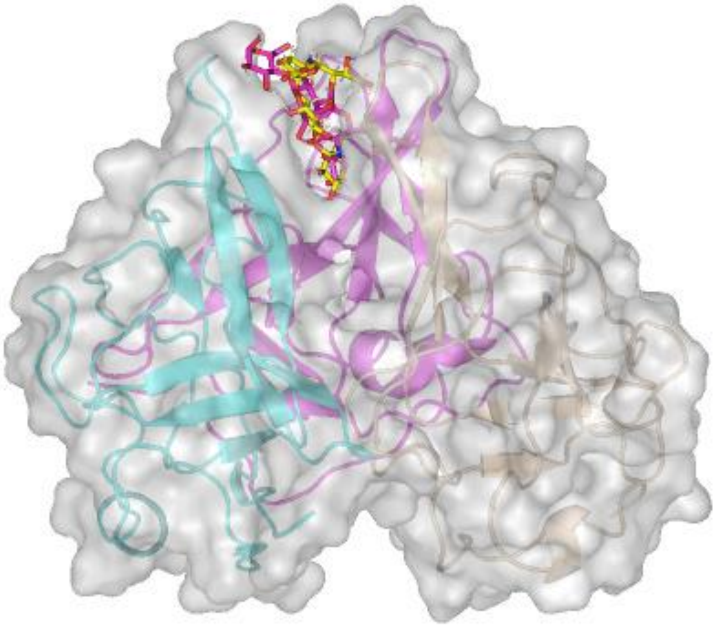

Supplementary Figure 19. Model of Neu5Ac-Lac binding to GNNV-P at pH 5.0.
Models of Neu5Ac-(α2,3)-Lac (magenta) and Neu5Ac-(α2,6)-Lac (yellow) binding to the GNNV-P trimer (shown as cartoon and surface representation).

Figure S20. Kyte-Doolittle hydrophobicity analysis

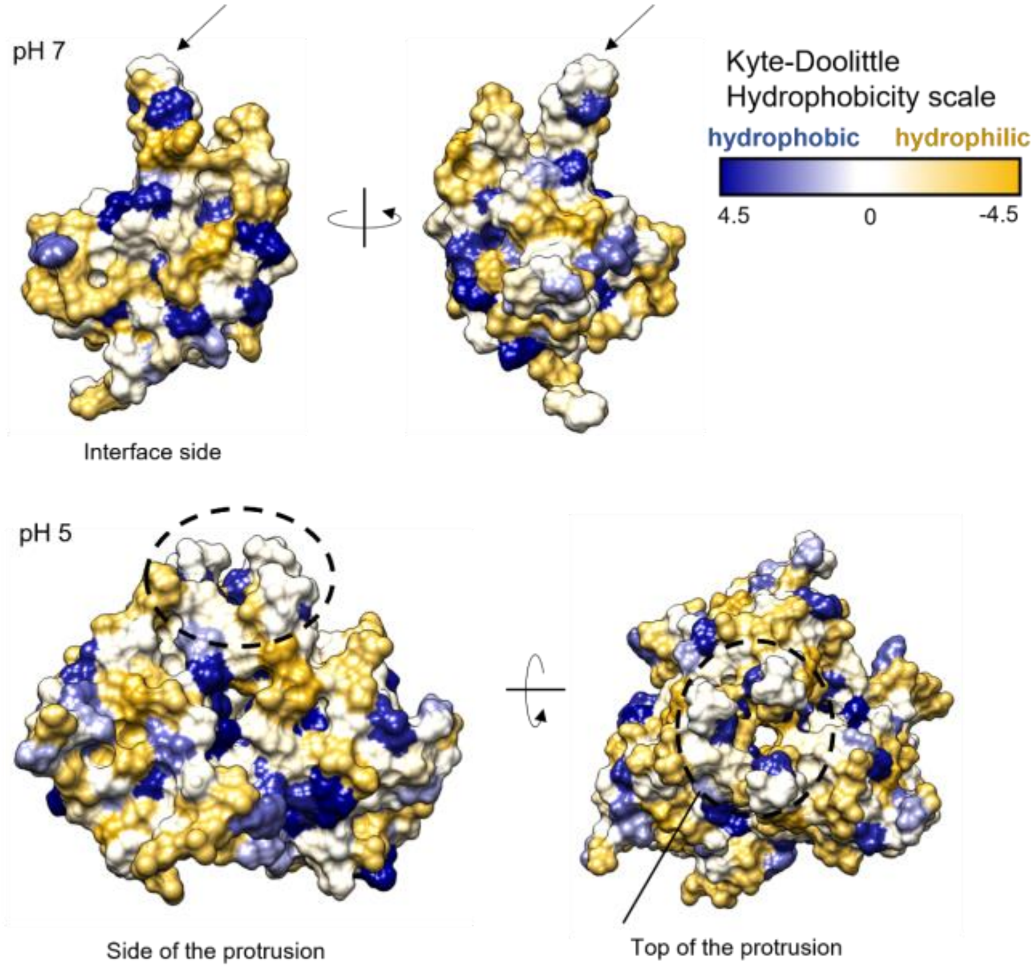

Supplementary Figure 20. Kyte-Doolittle hydrophobicity analysis

This Kyte-Doolittle analysis [28] shows distributions of hydrophobic and hydrophilic patches on P-domain surface. As shown in the figure, the tip of the trimer is not particularly hydrophobic; it is somewhat neutral (in white), i.e. in the middle between very hydrophobic and very hydrophilic. As three P-domains form a compact trimer, the tip patch in white color occupies a smaller area. This may alter the potential of NNV protrusion interacting with the endosomal membrane.

Figure S21. Flowchart of GNNV VLP image processing.

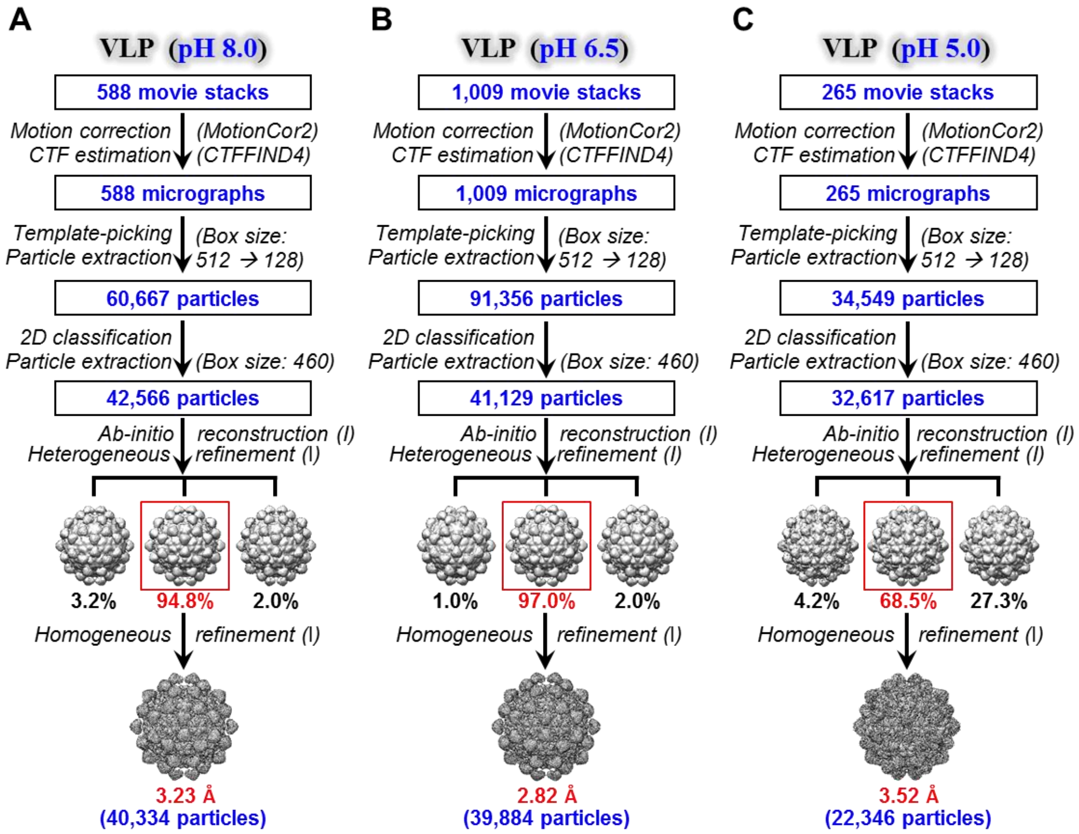

Supplementary Figure 20. Flowchart of GNNV VLP image processing.
(A) GNNV VLP at pH 8.0. **(B)** GNNV VLP at pH 6.5. **(C)** GNNV VLP at pH 5.0.

Figure S22. Flowchart of GNNV virion image processing.

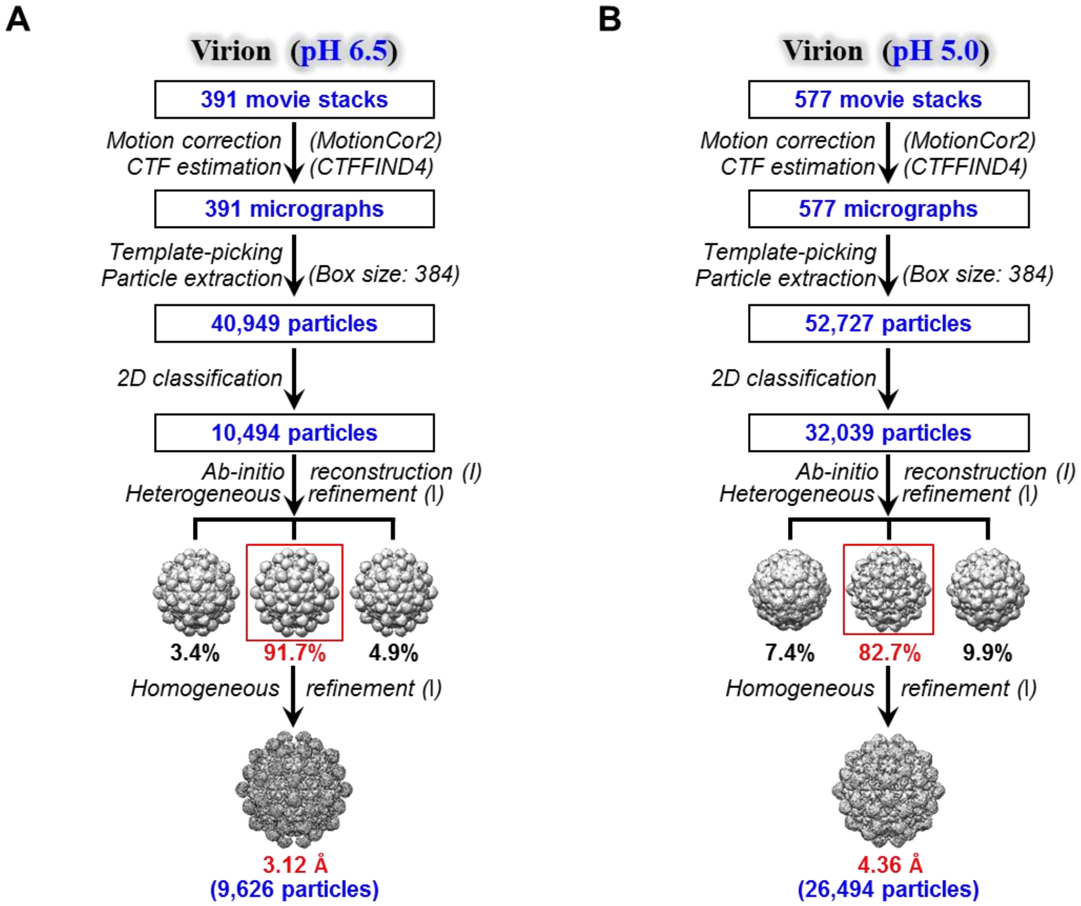

Supplementary Figure 21. Flowchart of GNNV virion image processing.
(A) GNNV VLP at pH 8.0. **(B)** GNNV VLP at pH 6.5. **(C)** GNNV VLP at pH 5.0.

Table S1. GNNV cryo-EM data collection, refinement, and validation statistics

	Virion (pH6.5)	Virion (pH5.0)	VLP (pH8.0)	VLP (pH6.5)	VLP (pH5.0)
**Data collection**					
EM equipment	Cryo-ARM	Cryo-ARM	JEM-2100F	FEI F-20	JEM-2100F
Voltage (kV)	200	200	200	200	200
Cs (mm)	1.4	1.4	3.3	2.3	3.3
Magnification (nominal)	40,000	50,000	50,000	29,000	50,000
Detector	K2	K2	DE-20	K2	DE-20
Detector (Operation mode)	Counting	Counting	Linear	Counting	Linear
Dose rate (e^-/Å ^{2} per second)	~ 6.5	~ 6.5	~ 20	~ 5	~ 20
Pixel size (Å)	1.36	1.09	1.16	1.24	1.16
Electron exposure (e^-/Å ^{2})	~ 52	~ 52	~ 30	~ 50	~ 30
Exposure time (s)	8	8	1.5	10	1.5
Frames (no.)	40	40	38	50	38
Defocus range (µm)	-0.45 ~ -3.05	-0.42 ~ -4.18	-0.57 ~ -2.76	-0.40 ~ -2.91	-0.87 ~ -2.93
**Reconstruction**					
Software	CryoSPARC (v2.0)	CryoSPARC (v2.0)	CryoSPARC (v4.3)	CryoSPARC (v4.3)	CryoSPARC (v4.3)
Micrographs stacks (no.)	391	577	588	1,009	265
Final particle images (no.)	9,626	26,494	40,334	39,884	22,346
Symmetry imposed	I	I	I	I	I
Map final resolution (Å) †	3.12	4.36	3.23	2.82	3.52
Map sharpening B-factor (Å ^{2})	-82.6	-197.8	-154.7	-110.5	-158.5
**Atomic modeling**					
Software	Coot & Phenix	-	Coot & Phenix	Coot & Phenix	Coot & Phenix
Number of protein residues #	509	-	509	509	873
Number of metal ions #	3 (Ca^{2+})	-	3 (Ca^{2+})	3 (Ca^{2+})	3 (Ca^{2+})
Number of atoms #	3,895	-	3,895	3,895	6,748
Map CC (around atoms) #	0.83	-	0.89	0.87	0.82
RMSD bond lengths (Å) #	0.004	-	0.005	0.005	0.005
RMSD bond angles (°) #	1.009	-	0.992	0.650	0.985
Clash score #	8.76	-	7.09	9.67	14.48

Ramachandran favored (%) #	96.22	-	96.62	97.42	96.08
Ramachandran allowed (%) #	3.78	-	3.38	2.58	3.92
Ramachandran outliers (%) #	0	-	0	0	0
Rotamer outliers (%) #	0	-	0	0	0
C_β deviations #	0	-	0	0	0
MolProbity score #	1.73	-	1.61	1.62	1.94

Statistics are given for one icosahedral asymmetric unit

†According to Fourier shell correlation (FSC)=0.143.

Table S2. Structural statistics of the 20 lowest energy GNNV-P structures.

**NMR restraints**	**GNNV-P (aa 221-336)**
Total distance constraints	853
Total NOE	765
Intra-residue	263
Inter-residue	502
Sequential ($\lvert i\text{-}j \rvert = 1$)	296
Medium-range ($1 < \lvert i\text{-}j \rvert < 5$)	71
Long-range ($\lvert i\text{-}j \rvert \geq 5$)	135
Hydrogen bond restraints	88
Dihedral angle restraints	
Φ	101
Ψ	101
Number of restraints per residue	8.4
Structure statistics	
Violations	
Distance violations per structure	
0.1-0.2 Å	0.8
0.2-0.5 Å	1.8
>0.5 Å	1.7
Dihedral angle restraints (>5°)	0
Ramachandran plot statistics (%) [a]	
Favored region	97.0
Allowed region	3.0
Outliers	0.0
Root Mean Square Deviation (RMSD) (Å) [b]	
Backbone	0.69 ± 0.13
Heavy atoms	1.62 ± 0.22

[a]wwPDB validation results (8XID)

[b]Pairwise RMSD of the ensemble of 20 strucutres was calculated in MOLMOL for secondary structure regions (α-helix: 240-243, 246-250; β-strands: 237-238, 256-259, 263-267, 275-285, 295-302, 307-310, 331-333).

Movie S1. Conformational change of GNNV VLP from pH 8.0 to 5.0.

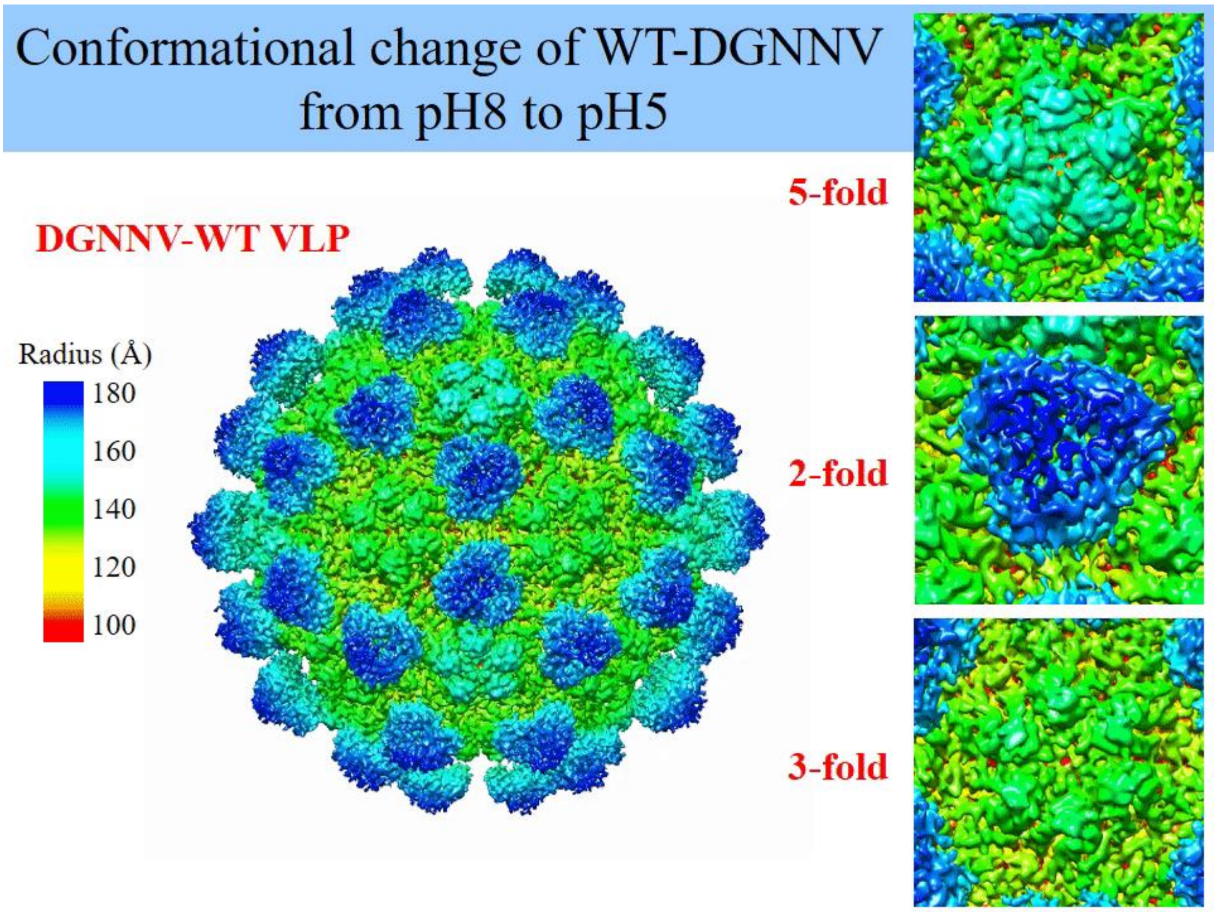

Movie S2. Enlarged views of GNNV protrusion conformational change from pH 8.0 to 5.0.

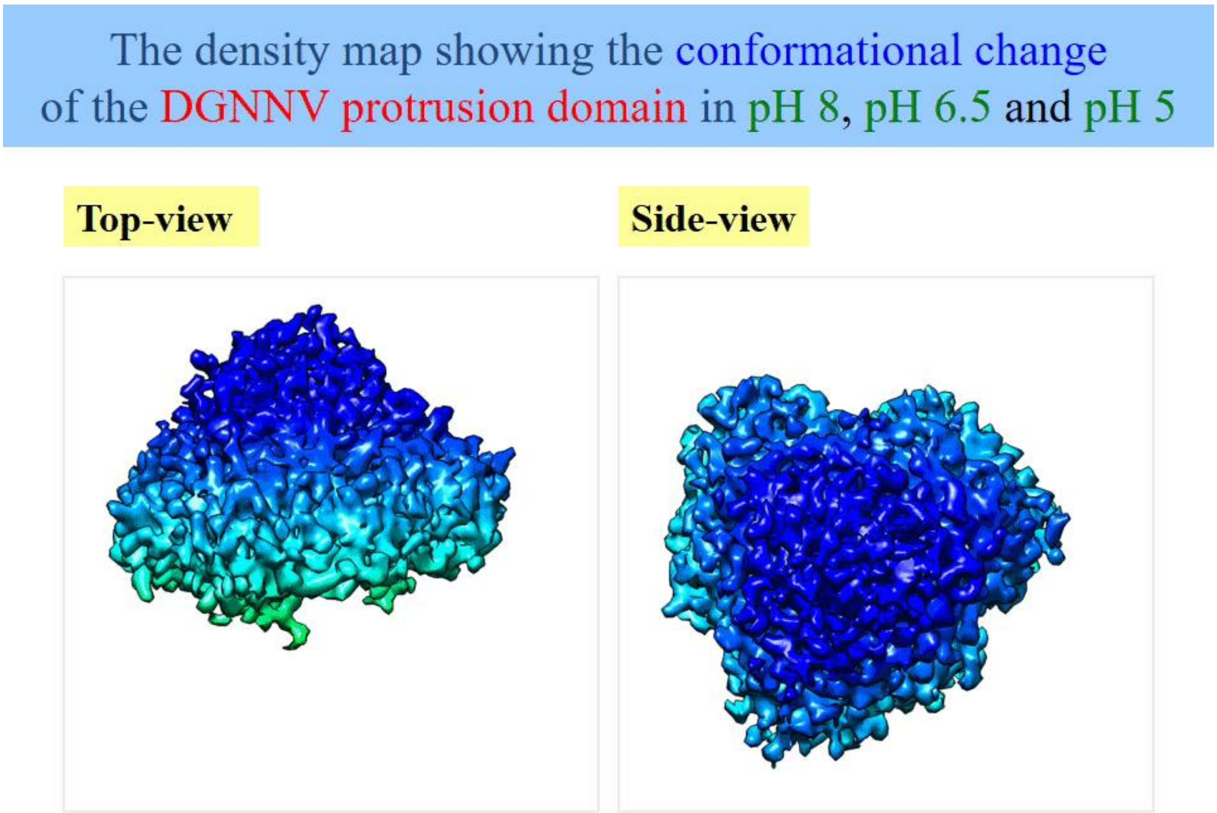

Movie S3. Hypothetical atomic model of GNNV-P conformational change from pH 8.0 to 5.0.

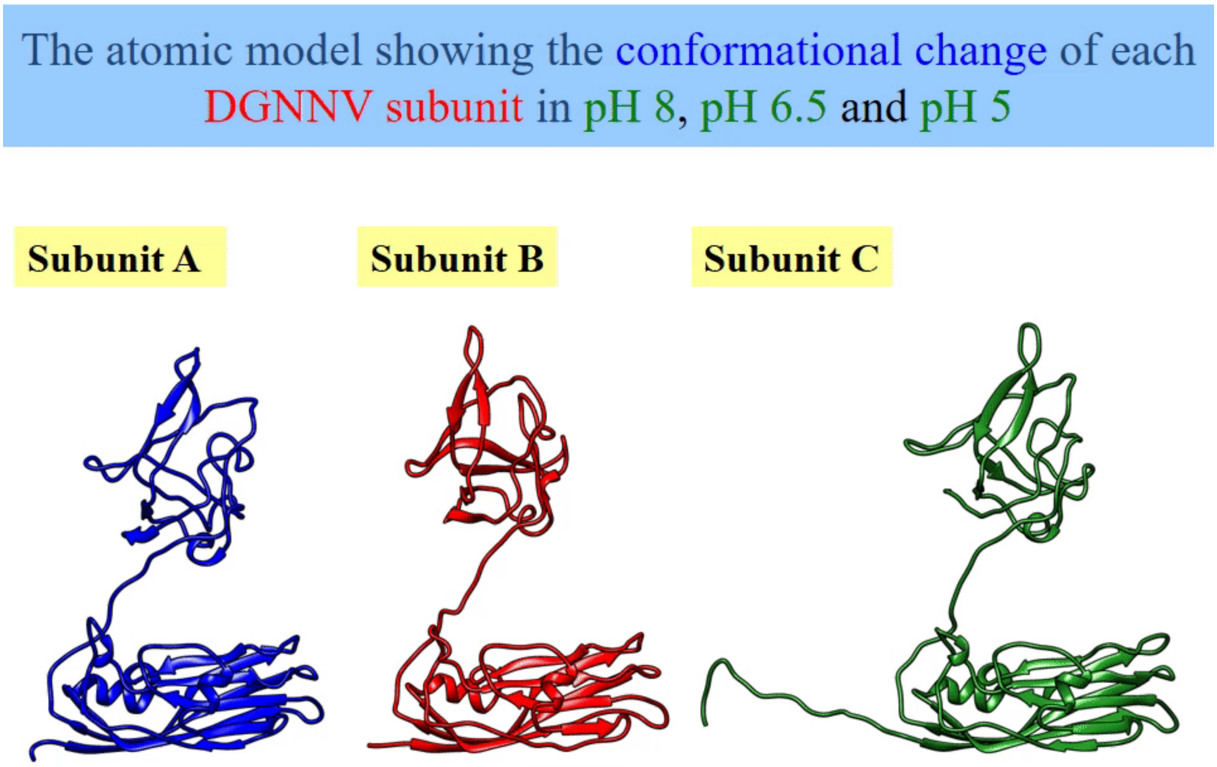

References

(1) Liu, W.; Hsu, C.-H.; Hong, Y.-R.; Wu, S.-C.; Wang, C.-H.; Wu, Y.-M.; Chao, C.-B.; Lin, C.-S. Early endocytosis pathways in SSN-1 cells infected by dragon grouper nervous necrosis virus. *Journal of General Virology* **2005**, 86 (9), 2553-2561.

(2) Wang, C. H.; Chen, D. H.; Huang, S. H.; Wu, Y. M.; Chen, Y. Y.; Hwu, Y.; Bushnell, D.; Kornberg, R.; Chang, W. H. Sub-3 Å Cryo-EM Structures of Necrosis Virus Particles via the Use of Multipurpose TEM with Electron Counting Camera. *Int J Mol Sci* **2021**, 22 (13).

(3) Zheng, S. Q.; Palovcak, E.; Armache, J. P.; Verba, K. A.; Cheng, Y.; Agard, D. A. MotionCor2: anisotropic correction of beam-induced motion for improved cryo-electron microscopy. *Nat Methods* **2017**, 14 (4), 331-332.

(4) Rohou, A.; Grigorieff, N. CTFFIND4: Fast and accurate defocus estimation from electron micrographs. *Journal of Structural Biology* **2015**, 192 (2), 216-221.

(5) Punjani, A.; Rubinstein, J. L.; Fleet, D. J.; Brubaker, M. A. cryoSPARC: algorithms for rapid unsupervised cryo-EM structure determination. *Nature Methods* **2017**, 14 (3), 290-296.

(6) Yang, Z.; Lasker, K.; Schneidman-Duhovny, D.; Webb, B.; Huang, C. C.; Pettersen, E. F.; Goddard, T. D.; Meng, E. C.; Sali, A.; Ferrin, T. E. UCSF Chimera, MODELLER, and IMP: an integrated modeling system. *J Struct Biol* **2012**, 179 (3), 269-278.

(7) Štěrbová, P.; Wu, D.; Lou, Y.-C.; Wang, C.-H.; Chang, W.-H.; Tzou, D.-L. M. NMR assignments of protrusion domain of capsid protein from dragon grouper nervous necrosis virus. *Biomolecular NMR Assignments* **2020**, 14 (1), 63-66.

(8) Dam, J.; Schuck, P. Calculating Sedimentation Coefficient Distributions by Direct Modeling of Sedimentation Velocity Concentration Profiles. In *Methods in Enzymology*, Vol. 384; Academic Press, 2004; pp 185-212.

(9) Ribaric, S.; Peterec, D.; Sketelj, J. Computer aided data acquisition and analysis of acetlycholinesterase velocity sedimentation profiles. *Computer Methods and Programs in Biomedicine* **1996**, 49 (2), 149-156.

(10) Johnson, B. A.; Blevins, R. A. NMR View: A computer program for the visualization and analysis of NMR data. *Journal of biomolecular NMR* **1994**, 4 (5), 603-614.

(11) Schwieters, C. D.; Kuszewski, J. J.; Marius Clore, G. Using Xplor–NIH for NMR molecular structure determination. *Progress in Nuclear Magnetic Resonance Spectroscopy* **2006**, 48 (1), 47-62.

(12) Maciejewski, M. W.; Schuyler, A. D.; Gryk, M. R.; Moraru, II; Romero, P. R.; Ulrich, E. L.; Eghbalnia, H. R.; Livny, M.; Delaglio, F.; Hoch, J. C. NMRbox: A Resource for Biomolecular NMR Computation. *Biophys J* **2017**, 112 (8), 1529-1534.

(13) Shen, Y.; Delaglio, F.; Cornilescu, G.; Bax, A. TALOS+: a hybrid method for predicting

protein backbone torsion angles from NMR chemical shifts. *J Biomol NMR* **2009**, 44 (4), 213-223.

(14) Tian, Y.; Schwieters, C. D.; Opella, S. J.; Marassi, F. M. A practical implicit solvent potential for NMR structure calculation. *J Magn Reson* **2014**, 243, 54-64.

(15) Gore, S.; Sanz García, E.; Hendrickx, P. M. S.; Gutmanas, A.; Westbrook, J. D.; Yang, H.; Feng, Z.; Baskaran, K.; Berrisford, J. M.; Hudson, B. P.; et al. Validation of Structures in the Protein Data Bank. *Structure* **2017**, 25 (12), 1916-1927.

(16) Williamson, M. P. Using chemical shift perturbation to characterise ligand binding. *Progress in Nuclear Magnetic Resonance Spectroscopy* **2013**, 73, 1-16.

(17) Schwarzinger, S.; Kroon, G. J.; Foss, T. R.; Wright, P. E.; Dyson, H. J. Random coil chemical shifts in acidic 8 M urea: implementation of random coil shift data in NMRView. *J Biomol NMR* **2000**, 18 (1), 43-48.

(18) Berendsen, H. J. C.; van der Spoel, D.; van Drunen, R. GROMACS: A message-passing parallel molecular dynamics implementation. *Computer Physics Communications* **1995**, 91 (1), 43-56.

(19) Jurrus, E.; Engel, D.; Star, K.; Monson, K.; Brandi, J.; Felberg, L. E.; Brookes, D. H.; Wilson, L.; Chen, J.; Liles, K.; et al. Improvements to the APBS biomolecular solvation software suite. *Protein Science* **2018**, 27 (1), 112-128.

(20) Martínez, L.; Andrade, R.; Birgin, E. G.; Martínez, J. M. PACKMOL: A package for building initial configurations for molecular dynamics simulations. *Journal of Computational Chemistry* **2009**, 30 (13), 2157-2164.

(21) Rühle, V. Pressure coupling / barostats. *Journal Club* **2008**.

(22) Essmann, U.; Perera, L.; Berkowitz, M. L.; Darden, T.; Lee, H.; Pedersen, L. G. A smooth particle mesh Ewald method. *The Journal of Chemical Physics* **1995**, 103 (19), 8577-8593.

(23) Lindorff-Larsen, K.; Piana, S.; Palmo, K.; Maragakis, P.; Klepeis, J. L.; Dror, R. O.; Shaw, D. E. Improved side-chain torsion potentials for the Amber ff99SB protein force field. *Proteins* **2010**, 78, 1950 - 1958.

(24) Mark, P.; Nilsson, L. Structure and Dynamics of the TIP3P, SPC, and SPC/E Water Models at 298 K. *The Journal of Physical Chemistry A* **2001**, 105 (43), 9954-9960.

(25) de Vries, S. J.; van Dijk, M.; Bonvin, A. M. J. J. The HADDOCK web server for data-driven biomolecular docking. *Nature Protocols* **2010**, 5 (5), 883-897.

(26) Xue, L. C.; Rodrigues, J. P.; Kastritis, P. L.; Bonvin, A. M.; Vangone, A. PRODIGY: a web server for predicting the binding affinity of protein-protein complexes. *Bioinformatics* **2016**, 32 (23), 3676-3678.

(27) Schuck, P.; Zhao, H. Sedimentation Velocity Analytical Ultracentrifugation: Interacting

Systems (1st ed.). CRC Press. 2017 https://doi.org/10.1201/b21988

(28) Kyte, J.; Doolittle, R. F. A simple method for displaying the hydropathic character of a protein. *J Mol Biol.* **1982**, 157(1):105-32.
